# Systems toxicology meta-analysis of *in vitro* assessment studies: biological impact of a candidate modified-risk tobacco product aerosol compared with cigarette smoke on human organotypic cultures of the aerodigestive tract†
†Electronic supplementary information (ESI) available. See DOI: 10.1039/c7tx00047b


**DOI:** 10.1039/c7tx00047b

**Published:** 2017-05-29

**Authors:** A. R. Iskandar, B. Titz, A. Sewer, P. Leroy, T. Schneider, F. Zanetti, C. Mathis, A. Elamin, S. Frentzel, W. K. Schlage, F. Martin, N. V. Ivanov, M. C. Peitsch, J. Hoeng

**Affiliations:** a PMI R&D , Philip Morris Products S.A. (part of the Philip Morris International group of companies) , Quai Jeanrenaud 5 , CH-2000 Neuchâtel , Switzerland . Email: julia.hoeng@pmi.com ; Fax: +41 (58)242 2811 ; Tel: +41 (58)242 2214; b Biology consultant , Max-Baermann-Str. 21 , 51429 Bergisch Gladbach , Germany

## Abstract

Systems biology combines comprehensive molecular analyses with quantitative modeling to understand the characteristics of a biological system as a whole. Leveraging a similar approach, systems toxicology aims to decipher complex biological responses following exposures. This work reports a systems toxicology meta-analysis in the context of *in vitro* assessment of a candidate modified-risk tobacco product (MRTP) using three human organotypic cultures of the aerodigestive tract (buccal, bronchial, and nasal epithelia). Complementing a series of functional measures, a causal network enrichment analysis of transcriptomic data was used to compare quantitatively the biological impact of aerosol from the Tobacco Heating System (THS) 2.2, a candidate MRTP, with 3R4F cigarette smoke (CS) at similar nicotine concentrations. Lower toxicity was observed in all cultures following exposure to THS2.2 aerosol compared with 3R4F CS. Because of their morphological differences, a smaller exposure impact was observed in the buccal (stratified epithelium) compared with the bronchial and nasal (pseudostratified epithelium). However, the causal network enrichment approach supported a similar mechanistic impact of CS across the three cultures, including the impact on xenobiotic, oxidative stress, and inflammatory responses. At comparable nicotine concentrations, THS2.2 aerosol elicited reduced and more transient effects on these processes. To demonstrate the benefits of additional data modalities, we employed a newly established targeted mass-spectrometry marker panel to further confirm the reduced cellular stress responses elicited by THS2.2 aerosol compared with 3R4F CS in the nasal culture. Overall, this work demonstrates the applicability and robustness of the systems toxicology approach for *in vitro* inhalation toxicity assessment.

## Abstract 2

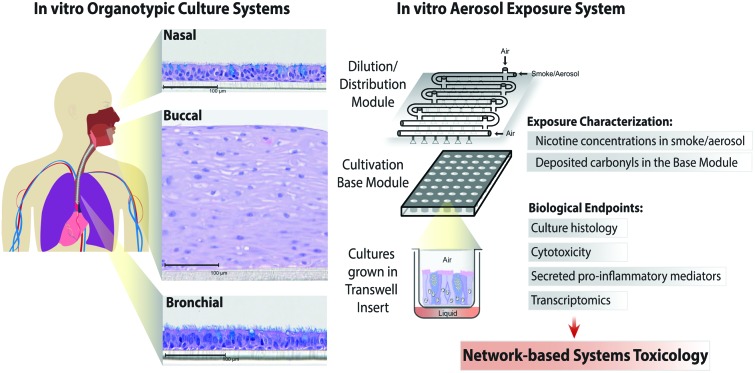
Reduced impact of a tobacco product was observed on the smoking “field-of-injury” tissues.

## Introduction

**Fig. 1:**
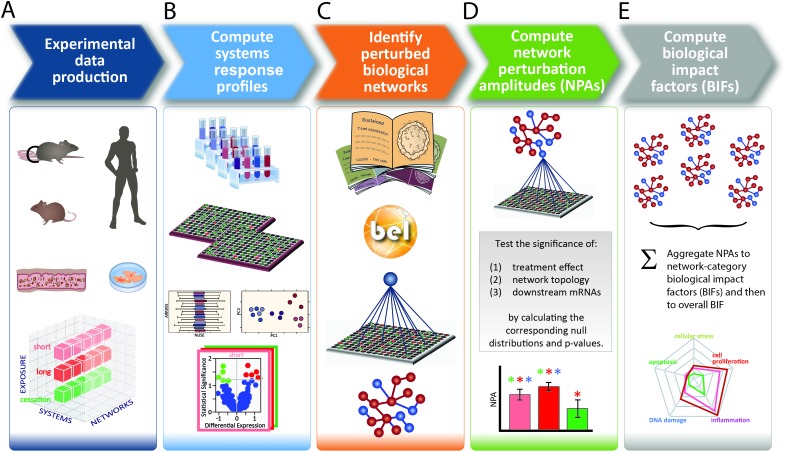
The causal network-based systems toxicology approach.

Toxicological analysis of complex mixtures, such as environmental exposures and cigarette smoke (CS), is challenging because toxicants affect biological systems through intricate interactions across various physiological processes. Using systems biology principles,^1^–^3^ systems toxicology integrates classical toxicology with advanced quantitative analyses of large datasets (including genomics, transcriptomics, proteomics, and metabolomics) across multiple levels of the biological organization to identify biological networks and molecular pathways affected by exposures [Fig. 1].^4^ This approach is well aligned with the 21^st^ Century Toxicology paradigm, because it can identify biological responses of toxicity pathways in human cells/tissues to infer adverse health outcomes in humans.^5^ Moreover, systems toxicology supports the shift in the focus of toxicological assessment from a hazard identification, in which high doses of chemicals are frequently tested in animal models, toward a safety-base paradigm, in which relevant doses of exposure—those sufficient to induce pathophysiological response typically occurring in the human population—are tested in appropriate cellular systems.

The pathophysiology of CS-induced effects in the human lung can be studied by obtaining biopsy samples. However, such an approach frequently involves invasive procedures (*e.g.*, bronchoscopy) and is limited to small amounts of samples. Although technologies have improved tremendously, for example, fibered confocal microscopy (“optical biopsy”) can be conducted during bronchoscopy,^6^,^7^ the available approaches do not allow to decipher the mechanisms of early toxicity impact on the field of injury.^17^ Alternatively, toxicological testing frequently relies on the use of animal models. However, strong efforts are being made by the scientific community—and further enforced by various regulatory bodies—to implement the 3Rs principle: to reduce, refine, and replace the use of animals.^8^ These efforts are equally motivated by the issue of cross-species translatability, in which results obtained from animal studies do not necessarily reflect the potential toxic impact in humans.

Organotypic culture models, which can be derived from human primary cells, are an alternative to investigate inhalation toxicity in human tissues. Unlike submerged monolayer cultures of respiratory epithelial cells, organotypic epithelium cultures are grown at the air–liquid interface and can therefore be directly exposed to CS, aerosols, or nanoparticles on the apical side.^9^,^10^ Following exposure to CS, tissue injury occurs not only in the distal lung, but also in the aerodigestive tract. In the oral cavity, CS exposure has been linked to inflammation and cancer.^11^–^14^ In response to CS exposure, the methylation and gene expression profiles of oral epithelial cells were similar to those of bronchial epithelial cells.^15^ In the nasal epithelium, the alteration of genes involved in xenobiotic metabolism—a biosensor of exposure—following smoking was reported to resemble that in the bronchial epithelium.^15^,^16^ Increased levels of the cytochrome P450 (CYP) 1A1 and 1B1 genes were also observed in both the nasal and bronchial epithelium of smokers, further supporting the relevance of nasal tissue as a surrogate lung airway epithelium.^17^ Various human organotypic culture models of the aerodigestive tract are available. Models derived from nasal or bronchial epithelial cells form a fully differentiated epithelium composed of basal, ciliated, and mucus-secreting goblet cells,^18^,^19^ while the models derived from buccal epithelial cells develop into a stratified non-keratinizing epithelium.^20^ Studies have demonstrated that the responses of these human organotypic cultures following CS exposure resemble those observed under *in vivo* situations.^21^–^24^ Recently, we have reported that these human organotypic culture models were useful and relevant to assess the effects of aerosol generated from the Tobacco Heating System (THS)2.2, a candidate modified risk tobacco product (MRTP), as compared with CS.^18^–^20^

Here, we report on a systems toxicology meta-analysis of the biological impact of exposure to THS2.2 aerosol and 3R4F CS on three types of organotypic cultures of aerodigestive epithelia (leveraging data from our recent publications: buccal,^20^ nasal,^18^ and bronchial^19^). This work further explores whether the “field of injury” concept^15^—CS exposure affects similar mechanisms across the entire tissues lining the aerodigestive tract—can be reproduced in these *in vitro* studies. In the context of such a meta-analysis, this work demonstrates how the systems toxicology framework enables a comprehensive toxicological and mechanistic assessment across tissues and exposure conditions. Overall, the data show that 3R4F CS exposure triggers similar mechanisms in all three tissues, demonstrating an *in vitro* observation of the “field of injury.” This work also reveals that the reduced levels of harmful and potentially harmful chemicals in the THS2.2 aerosol compared with the 3R4F smoke^25^ translate into reduced perturbations of critical biological processes including inflammation and cellular stress that were consistently affected by 3R4F CS exposure across the three *in vitro* human organotypic airway epithelium cultures.

## A causal biological network enrichment approach for systems toxicology-based assessment

A five-step systems toxicology assessment strategy has been proposed.^26^–^28^ This strategy starts with the production of experimental data based on a robust experimental design, in which relevant exposure conditions and biological test systems are specified, along with a sufficient number of biological replicates (Fig. 1A). Omics technologies are leveraged to comprehensively capture the molecular impact of exposures as System Response Profiles; in the case of transcriptomic data, they correspond to global gene expression changes between exposed and unexposed control samples (Fig. 1B). To provide quantitative mechanistic insights into exposure effects based on transcriptomic data, a network enrichment approach has been developed (Fig. 1C).^26^–^28^ The approach relies on a collection of toxicologically relevant Causal Biological Network Models.^29^ These networks consist of literature-supported cause-and-effect relationships between molecular entities that are encoded in the Biological Expression Language (BEL, ; http://openbel.org/). The relationships are directional, and thus may describe enzymatic activation, gene transcript up-regulation, or protein complex formation. A molecular entity within these networks can represent mRNA abundance, enzymatic activity of a protein, and activity of a biological process, and can be quantified. The Causal Biological Network Models are hierarchically organized, computable, and context-relevant. Concretely, our causal network collection, which was developed specifically for respiratory toxicology assessments, contains toxicologically relevant networks in five categories:^29^ cellular stress (*Cell Stress* (CST) network), inflammation (*Inflammatory Process* network (IPN)), cellular proliferation (*Cell Proliferation* (CPR) network), cellular repair and angiogenesis (*Tissue Repair and Angiogenesis* network), and cellular fate (*Cell Fate* (CFA) network). The development of this network collection involved a multistep process to ensure their biological and scientific relevance. A team of discipline-specific experts first defined relevant boundaries for each network and subsequently conducted literature searches to identify relevant causal relationships in the literature within these boundaries.^30^–^34^ Thereafter, a data-driven approach was conducted using relevant context-specific datasets to augment the initial literature-based BEL-encoded networks.^35^ Finally, a crowd-sourcing approach was taken to confirm the appropriateness of the network models.^36^ Combining the System Response Profiles and these causal network models, a network enrichment algorithm (the network perturbation amplitude (NPA)) is used to quantify the biological impact of an exposure on a given network (Fig. 1D). The approach, thus, integrates the systems response profiles in the context of these causal network models.^27^,^28^ Unlike the more commonly used gene set overrepresentation and gene set association approaches—*e.g.*, gene set enrichment analysis^37^ in which the structure of the biological network is not taken into account—the NPA methodology explicitly considers the network structure in its enrichment score. Thus, the NPA method falls into the emerging category of Pathway Topology algorithms.^38^ Furthermore, the NPA methodology directly provides an amplitude for a treatment-induced effect rather than an abstract score or a simple *p*-value. Three statistics are used to assess the statistical significance of a network perturbation: (1) a 95% confidence interval for the significance of the treatment effect; (2) a specificity *p*-value obtained for randomized versions of the network; and (3) a specificity *p*-value obtained for randomly assigned downstream mRNA transcripts.^21^ Finally, the network-level NPA scores are aggregated by network categories,^21^,^39^ making use of the hierarchical organization of the Causal Biological Network Models (Fig. 1E). Overall, the network-based systems toxicology approach provides not only a quantitative assessment but also a mechanistic insight into the affected biological processes, thus going beyond traditional toxicogenomics that often rely on a non-targeted interpretation of differential gene expression.

## Materials & methods

### Buccal, bronchial, and nasal datasets

**Fig. 2:**
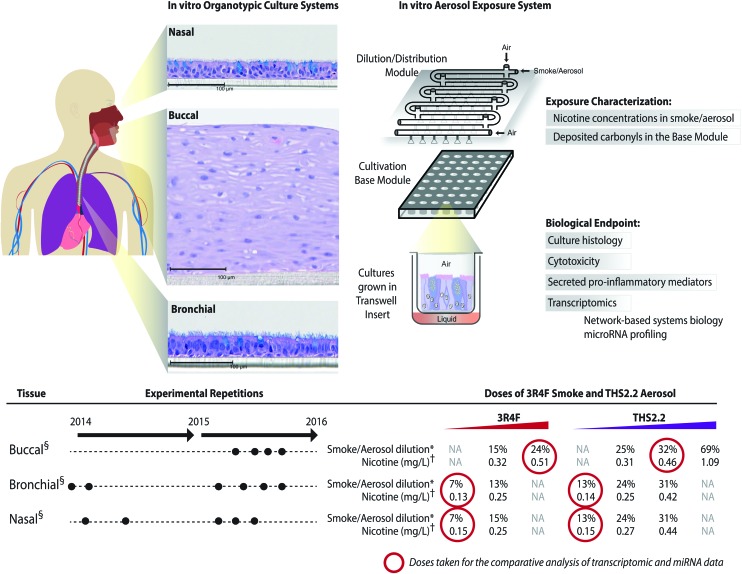
A series of *in vitro* studies using human organotypic epithelium cultures. Organotypic culture models recapitulating the human aerodigestive tract lining the “tissue of injury” fields (buccal, bronchial, and nasal) were exposed to 3R4F CS or THS2.2 aerosol at similar nicotine concentrations in an Exposure System (Vitrocell 24/48®). An illustration of the air–liquid interface organotypic culture located in the Base Module of the exposure system is shown. Exposure characterization throughout the study period included measurements of nicotine concentrations (in the CS/aerosol) and deposited carbonyl concentrations (in the phosphate-buffered saline-filled Cultivation Base Module). Various biological endpoints were measured at various post-exposure time points as illustrated. The detailed experimental procedures have been described previously.^18^–^20^  Three independent exposure-runs were conducted for each item (3R4F and THS2.2); except for those using bronchial cultures in 2014. * Dilution refers to the percentage 3R4F smoke or THS2.2 aerosol diluted with air in the Dilution/Distribution Module of the Exposure System. † Nicotine concentration (mg L^–1^) refers the corresponding concentration to the specific dilution of smoke/aerosol determined by trapping the diluted smoke/aerosol in the EXtrelut® 3NT column. § The nasal organotypic cultures were reconstituted from the primary nasal epithelial cells of 30 year-old non-smoker male; buccal organotypic cultures were reconstituted from the primary buccal epithelial cells of 46 year-old non-smoker male; and bronchial organotypic cultures were reconstituted from the primary bronchial cells of 28 year-old non-smoker male (except for the first two experimental repetitions in which the bronchial cultures were reconstituted from 23 year-old non-smoker male). NA: not available.

The meta-analysis of the biological impact of exposure to THS2.2 aerosol and 3R4F CS was conducted leveraging data from our recent publications on three organotypic cultures of aeroddigestive tract epithelia: buccal,^20^ nasal,^18^ and bronchial.^19^ For each culture type, experimental repetitions were done in which on average 3 independent exposure runs were performed (Fig. 2). The detailed experimental procedures and protocols (*i.e.*, exposure procedures, adenylate kinase (AK) release assay, and Luminex-based analysis of the pro-inflammatory mediators) have been described previously: buccal;^20^ bronchial;^19^ and nasal.^18^ The transcriptomic dataset used in this present work is available in Array Express (; http://www.ebi.ac.uk/arrayexpress/) with the following IDs: E-MTAB-4742 (buccal dataset); E-MTAB-5179 (bronchial dataset); and E-MTAB-4740 (nasal dataset).

### Ciliary beating functionality of the ciliated pseudostratified epithelium (bronchial and nasal cultures)

Video recording of the beating cultures (bronchial/nasal) was performed before exposure, immediately after (0 h) and 4 h, 24 h, 48 h, and 72 h after exposure using a digital high-speed video camera (Sony CXD V60, Sony, Tokyo, Japan) connected to an inverted microscope system (Leica DMi8, Leica, Wetzlar, Germany) at a rate of 90 frames per second as previously described.^18^ The ciliary beating functionality was evaluated by four measures: the weighted frequency, the uniformity of the detected frequency, the active area, and the power of the detected signal (Fast Fourier Transformation (FFT)). The methodologies of the analysis are different from those reported in our previous publications.^18^,^19^ The analysis was done on a total of 512 video frames recorded from the center of the insert surface. For each pixel, the mean of the 512 frames was subtracted, and subsequently a FFT and an approximate Barlett's Kolmogorov–Smirnov test were performed on the pixel intensity. To calculate the weighted frequency, the mean of the dominant frequency detected was weighted by its FFT power magnitude for each video if the pixel was active (*p* ≤ 0.001) and its dominant frequency was in the range of 0–20 Hz. To measure the uniformity of the detected frequency, the mean of the Kolmogorov–Smirnov statistics was used; *i.e.*, the maximum difference of the normalized cumulative FFT spectrum and the uniform cumulative distribution function. The active area was defined as the proportion of pixels that show an unadjusted Bartlett's Kolmogorov–Smirnov *p*-value ≤0.001. Finally, the strength of the ciliary beating signal was estimated as the sum of the FFT power spectra in the range 2.5–20 Hz.

### Targeted proteomics by parallel-reaction monitoring

Targeted proteomics by parallel-reaction monitoring (PRM) was conducted at the 48 h post-exposure time point for the nasal organotypic cultures that were exposed to 3R4F CS (0.15 mg nicotine per L), THS2.2 aerosol (0.15 mg L^–1^, 0.27 mg L^–1^, and 0.44 mg nicotine per L), and fresh air as described by Iskandar *et al.*^18^ Twelve experimental replications (separate exposures) were analyzed for 3R4F CS and THS2.2 aerosol-exposed samples, each paired with the air-exposed samples in each experimental replication.

Soste *et al.* have demonstrated how targeted proteomic analysis of sentinel proteins, *i.e.* biological marker proteins, can efficiently capture the activation state of up to 188 biological processes in baker's yeast.^40^ Guided by this idea, a protein marker panel (sentinel panel) was defined to cover the major effect categories relevant to organotypic exposure studies: xenobiotic metabolism, oxidative stress, metabolic adaptations, unfolded-protein response (UPR), tissue composition changes, barrier function, and senescence (Fig. 9). The list of targeted proteins for these effect categories together with details of the spiked-in peptides is presented in ESI Table 1.†

Total protein was extracted from the cultures using a commercially available sample preparation kit (Biognosys AG, Schlieren, Switzerland). The cells were incubated in denaturing buffer and disrupted by ultrasound treatment. The protein concentration was determined using a Pierce 660 nm protein assay (Thermo Scientific). Fifty microgram of protein was subjected to protein reduction, alkylation, and digestion as described in the manual. Prior to analysis, samples were purified using C18 reversed phase and solid phase extraction plates (Waters). After resuspension in LC-Buffer A (1% acetonitrile, 0.1% formic acid), stable-isotope labeled reference peptides were spiked into the sample for each target of interest. About 0.5 μg of total protein was injected for analysis onto a 15 cm C18 reversed-phase column and analyzed by liquid chromatography coupled with tandem mass spectrometry using an Easy-nano LC 1000 instrument connected online to a Q-Exactive Plus (System 1) or Q-Exactive HF mass-analyzer (System 2, both from Thermo Scientific).

On system 1, peptides were separated using a PepMap RSLC C18 column (50 μm × 15 cm, 2 μm particle size, 100 Å pore-size, Thermo Scientific) with a flow rate of 200 nL min^–1^ in a gradient starting with 5% LC-Buffer B (95% acetonitrile, 0.1% formic acid) to 28% LC-Buffer B over 50 min followed by a 10 min column wash step at 100% LC-Buffer B. The Q-Exactive Plus mass spectrometer was operated in retention time scheduled PRM mode with a resolution of 17.500, an AGC target value of 1e6, a maximum injection time of 30 ms and a precursor isolation window of 1.2 *m*/*z*.

On system 2, peptides were separated using an Acclaim PepMap RSLC C18 column (75 μm × 15 cm, 2 μm particle size and 100 Å pore-size, Thermo Scientific) with a flow rate of 300 nL min^–1^ in a gradient of 5% LC-Buffer B to 30% LC-Buffer B over 30 min followed by a 10 min column wash step at 100% LC-Buffer B. A Q-Exactive HF mass spectrometer was operated in retention time scheduled PRM mode with a resolution of 30 000, an AGC target value of 1e6, a maximum injection time of 60 ms and a precursor isolation window of 1.2 *m*/*z*. For retention time scheduling, iRT peptides (Biognosys AG, Schlieren, Switzerland) were used (Escher *et al.*, 2012)^95^.

Raw files from the PRM acquisition were analyzed with SpectroDive (version 7.5, BiognoSYS AG). Ion chromatograms for the endogenous peptides and the corresponding stable isotope labeled reference peptides were extracted for all the measured transitions (ESI Table 1†) using the software vendor's default settings. For quantification, the area under curve (AUC) intensities of all transitions were summed and the ratios of AUC sums of the endogenous and corresponding reference peptide signals were calculated.

For the statistical analysis, a linear model was fitted for each exposure and the respective sham group, including the experimental repetition as a covariate to account for the pairing between exposure and sham groups.^41^ The obtained raw *p*-values (without empirical Bayes moderation, corresponding to a paired *t*-test) were adjusted across protein markers using the Benjamini–Hochberg false discovery rate (FDR) method. Differentially expressed proteins were defined as those with a FDR-adjusted *p*-value of <0.05.

### Computational analyses

For comparative systems toxicology assessment using the causal network approach, a collection of 29 (bronchial/nasal) or 28 (buccal) causal biological networks relevant for organotypic epithelium cultures were used (ESI Table 2†).^29^ Using these network models and gene differential expression values, we applied our Causal Biological Network Enrichment Approach to calculate network perturbation amplitudes (NPA) and biological impact factors to quantify the systems responses to the various exposures^28^ [Fig. 1].

In parallel, a gene set analysis (GSA) was performed with pathway maps from the KEGG knowledge base.^42^ The significance of the gene-set enrichment was assessed using a competitive null hypothesis (Q1) and a self-contained null hypothesis (Q2).^43^ Whereas Q1 tests for the significance of genes in the set *versus* those not in the set, Q2 tests for a significant difference between the conditions. With this, we expect Q2 to be more appropriate in the context of comparative toxicity assessment (*e.g.*, to reveal a significant effect on a given gene set compared with air-exposed controls exposure), while Q1 can highlight gene-sets that dominate these responses. Here, the Q1 statistics were calculated with the Camera approach^44^ and the Q2 statistics with the Roast approach,^45^ which take the gene correlation structures into account. The resulting *p*-values were adjusted for multiple hypothesis testing using the Benjamini–Hochberg procedure.

The comparability across the exposure-induced biological impact was examined using clustering with two distinct network-based similarity metrics (amplitude-based and shape-based). The amplitude-based metric considers two exposure conditions with similarly high NPA values to be closely related. To calculate this metric, first all the node-level contributions to the normalized NPA values^28^ were concatenated across all networks; these values were normalized so that, for all networks, the maximal normalized NPA value across all exposure conditions was 1, and so that all the node contributions from not statistically significantly perturbed networks were 0; finally, the Euclidean distance matrix between the available 24 considered exposure conditions was calculated. The “shape-based” metric specifically compares the relative distributions of the node-level contributions across exposure conditions. Two exposure conditions with similar distributions of their node-level contributions (but not necessarily the same magnitude of non-normalized NPAs) are considered to be closely related. To calculate this metric, it was assumed that the normalized node contributions for any given network and exposure conditions summed to 1; then the corresponding distance matrix was calculated using the Manhattan formula, which is appropriate for comparing concatenated (normalized) distributions. For clustering, an affinity propagation-based approach was applied in two steps to identify clusters and the connections between their “exemplar elements”.^46^ The defined graph encodes the similarities between exposure conditions within and between the obtained clusters.

## Results and discussion

### Exposure impact on cytotoxicity and ciliary beating functionality

The human organotypic epithelium cultures (buccal, bronchial, and nasal) had been acutely exposed (for 28 min) to 3R4F CS at two nicotine concentrations: the low dose of 3R4F CS was matched by nicotine concentration to the low dose of THS2.2 aerosol, and the high dose of 3R4F CS was matched by the medium dose of THS2.2 aerosol.^18^–^20^ In addition, a high dose of THS2.2 aerosol was tested at a nicotine concentration approximately threefold the low dose of 3R4F CS (Fig. 2). Because of the morphological differences across the organotypic cultures (Fig. 2)—*e.g.*, the thickness of the buccal cultures (stratified epithelium) was approximately five times that of the bronchial/nasal cultures (pseudostratified epithelium)—the buccal cultures were exposed to higher doses of 3R4F CS and THS2.2 aerosol (the selection of the doses has been described before^20^). To increase the robustness of the assessment, for each culture type, a series of experimental repetitions were conducted (Fig. 2). For each experimental repetition, an average of three independent exposure runs were performed.

**Fig. 3:**
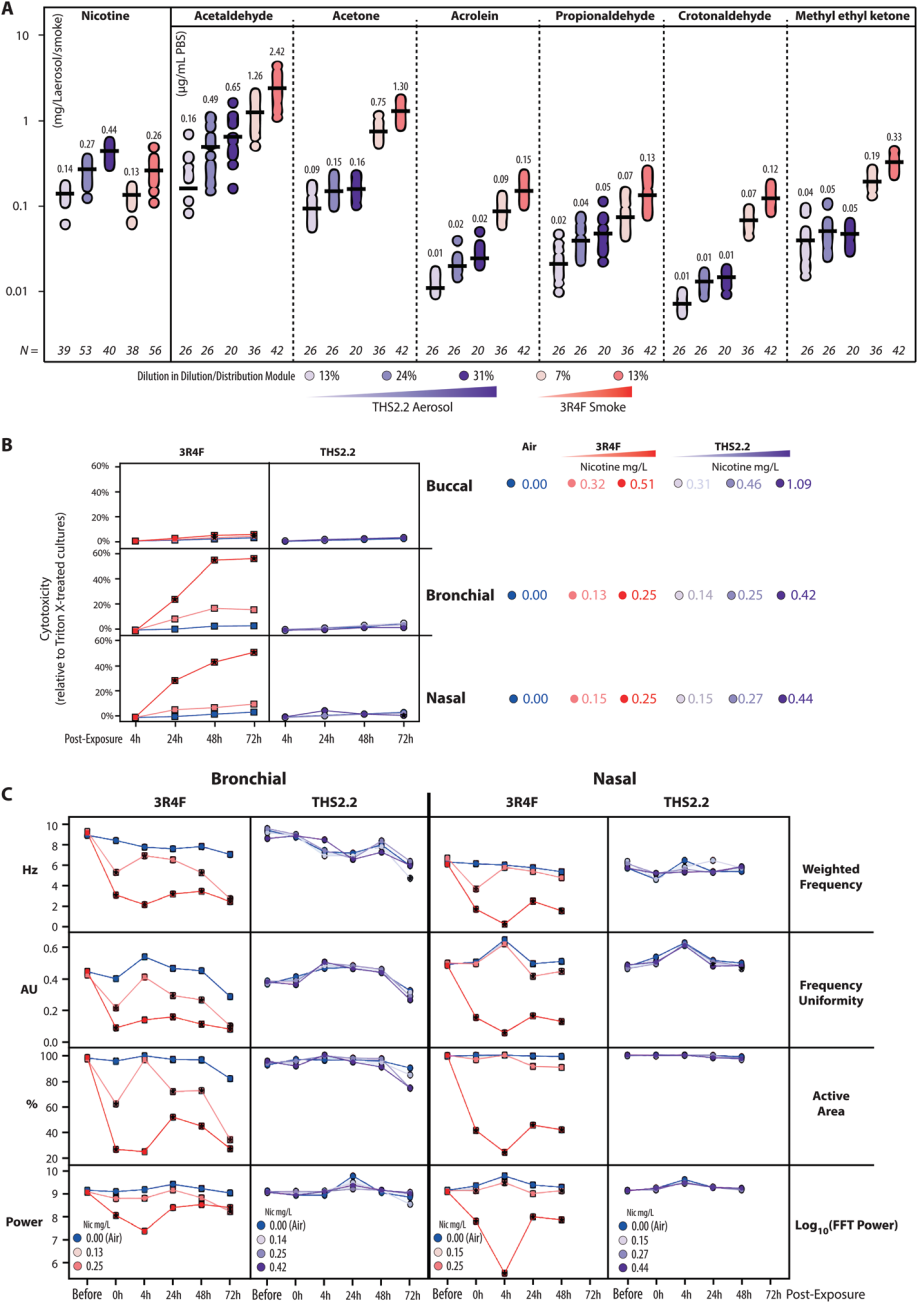
Characterization of the 3R4F CS/THS2.2 aerosol in the exposure system and assessment of cytotoxicity and ciliary beating. (A) Concentrations of nicotine in the diluted 3R4F CS and THS2.2 aerosol (mg nicotine per L) were measured by trapping the diluted smoke/aerosol in EXtrelut® columns and detection by gas chromatography-flame ionization (samplings were done throughout the study period). In addition, the concentrations of deposited carbonyls in the PBS-filled Cultivation Base Module of the exposure system were determined. (B) Cytotoxicity levels following exposure were measured based on the levels of adenylate kinase activity in the basolateral media (adenylate kinase released assay) at various post-exposure time points in the buccal, bronchial, and nasal cultures. (C) Ciliary beating functionality of the ciliated pseudostratified epithelium (bronchial and nasal) cultures was assessed longitudinally before, immediately after (0 h) and 4 h, 24 h, and 48 h after exposure (as well as 72 h for the bronchial culture only). Weighted frequency (Hz) is the mean frequency over the pixel, weighted by the FFT power at the pixel dominant frequency. Frequency uniformity (arbitrary unit, AU) is an index expressing the distribution of the detected frequency in the FFT spectrum (0 = blank noise; 1 = unique frequency). The active area (%) is the percentage the detected pixels differ from the blank noise. The log_10_(FFT Power) is an estimate of the detected power based on the beating signal of the ciliary movement.

Characterization of the 3R4F CS and THS2.2 aerosol in the exposure system (Vitrocell 24/48®) included the measurements of nicotine—previously described^47^—in the 3R4F CS and THS2.2 aerosol throughout the studies. The measurements were done to assess the reproducibility of the nicotine concentrations for a given dilution of 3R4F CS or THS2.2 aerosol. Fig. 3A shows that reliable nicotine concentrations were achieved for a particular smoke/aerosol dilution, despite some variability (descriptive statistics results are given in ESI Table 3†). Moreover, the concentrations of carbonyls deposited in the Cultivation Base Module were also determined throughout the studies (Fig. 3A) and were found to be comparable across the exposure experiments for the particular doses of 3R4F CS and THS2.2 aerosol (descriptive statistics results are given in ESI Table 3†). The concentrations of the deposited carbonyls remained lower in the PBS samples exposed to THS2.2 aerosol compared with 3R4F CS at all the tested doses although the nicotine concentrations between THS2.2 and 3R4F CS were similar (*i.e.*, the nicotine concentration in the low dose of 3R4F CS was comparable to the low dose of THS2.2; and the high dose of 3R4F CS was comparable to the medium dose of THS2.2). A more extensive characterization of the THS2.2 aerosol has been reported previously.^25^

Acute 3R4F CS exposure (at 0.13–0.15 mg nicotine per L) elicited a similar cytotoxicity profile (based on the level of adenylate kinase released into the basolateral media upon cell damage) in both the bronchial and nasal cultures: the cytotoxicity increased dose dependently and with the duration of post-exposure (Fig. 3B). Even at higher doses (nicotine concentrations of 0.32–0.51 mg nicotine per L), 3R4F CS elicited a less pronounced cytotoxicity in the buccal cultures compared with the bronchial and nasal cultures following exposure to 3R4F CS at 0.25–0.27 mg nicotine per L. The 3R4F CS-induced cytotoxicity in the buccal cultures increased only slightly (maximum ∼6%) with the post-exposure duration. This cytotoxicity measurement relies on the level of adenylate kinase in the basolateral media; therefore, its level in the apical compartment was not taken into account that could potentially underestimate the cytotoxicity measurement—in particular, for a thick stratified epithelium culture. Nevertheless, a histological assessment of the buccal cultures demonstrated some detachment above the basal cell layer, keratinization/desquamation of the epithelium, and the presence of intracellular granular structures (reported previously^20^). In contrast, THS2.2 aerosol exposure elicited only minimal cytotoxicity in all three cultures at all the doses and post-exposure time points tested.

An additional functional parameter was inferred from the ciliary beating functionality of the ciliated pseudostratified epithelium airway cultures (bronchial and nasal) following exposure. Mucociliary clearance—a mechanism driven by the coordinated movement (beating) of cilia to transport mucus-containing toxicants in the respiratory tract^48^—is an initial defense mechanism to clear inhaled toxicants.^49^Fig. 3C (1^st^ row, Weighted Frequency by the power of the beating signal) shows that 3R4F CS exposure (at 0.25 mg nicotine per L) reduced the frequency of the ciliary beating in both bronchial and nasal cultures immediately after exposure. The weighted frequency remained low at the later post-exposure time points compared with the air control. Fig. 3C (2^nd^ row, Frequency Uniformity) shows that 3R4F CS exposure (at 0.25 mg nicotine per L) interrupted the uniformity of the beating frequency in both bronchial and nasal cultures. Less uniformity of the beating frequency was observed at all post-exposure time points, which suggested that the normal propulsion of the mucus layer was disrupted following 3R4F CS exposure. Both disorganization of the cilia beating frequency and reduction in ciliary beating frequency have been reported in individuals with asthma.^48^ Moreover, in both bronchial and nasal cultures exposed to 3R4F CS (at 0.25 mg nicotine per L), the areas at which active ciliary beating was detected were reduced (Fig. 3C, 3^rd^ row, Active Area). The smaller active area could be attributed to the loss (shedding) or shortening of the cilia, similarly to what has been observed in nasal biopsies of individuals exposed to high levels of air pollution.^50^ The power of the detected signal (log FFT power, Fig. 3C, 4^th^ row) further confirmed that 3R4F CS exposure impacted the beating signal immediately after exposure (particularly for the 3R4F CS at 0.25 mg nicotine per L). The overall data further reveal that the cultures have the capacity to recover following exposure: the power of the beating signals eventually returned to levels comparable to the air-exposed samples (at the 24 h and 48 h post-exposure time points, and 72 h for the bronchial cultures). However, the weighted frequency, frequency uniformity, and active area remained low. The observation suggests that despite recovering (regaining power of the beating signals), the beating frequency and the coordinated ciliary movement remained compromised. Moreover, the analysis demonstrated a distinct profile of the ciliary beating function following exposure: a dose-dependent impact of 3R4F CS was observed in the bronchial cultures but not in the nasal cultures. This suggests that the nasal cultures exhibit superior defense capabilities (with regard to mucociliary clearance), which is consistent with the role of the nasal epithelium as the first lines of defense against inhaled pathogens, dusts, and irritants.^51^ Conversely, exposure to THS2.2 aerosol at all the tested concentrations did not impact the weighted ciliary beating frequency, the uniformity of the frequency, the active area, or the power of the beating signal. The findings were similarly observed in both bronchial and nasal cultures, further supporting the minor cytotoxicity impact of THS2.2 aerosol discussed earlier.

### Exposure-induced perturbation of molecular mechanisms: a comparative analysis of the buccal, bronchial, and nasal transcriptomes

**Fig. 4:**
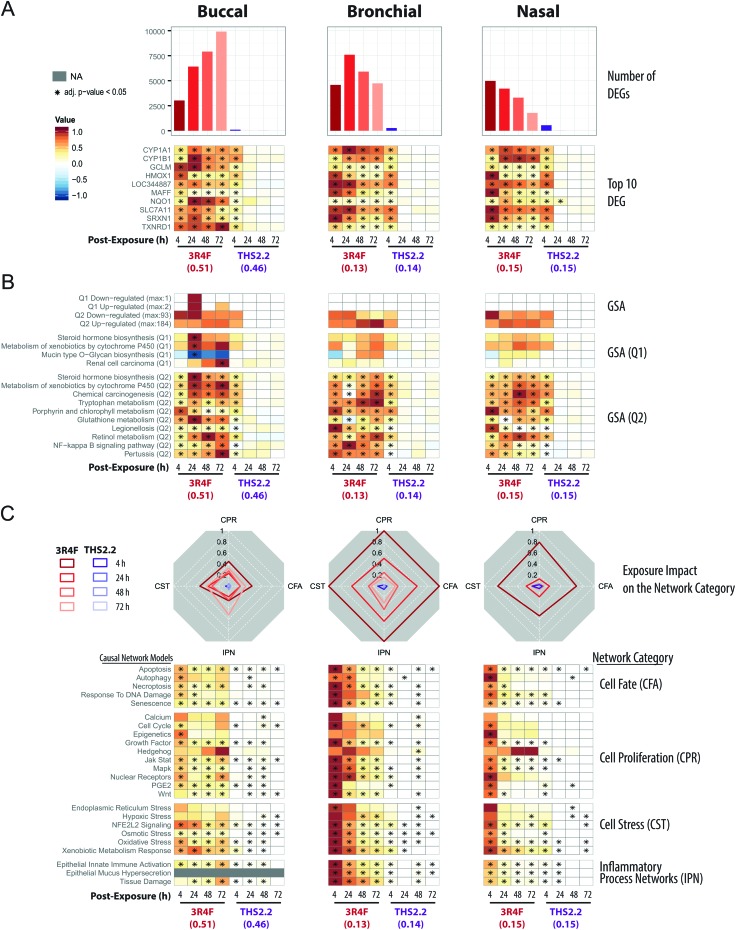
Mechanistic investigation of the exposure impact based on the transcriptomic data. (A) Barplots showing the number of significantly differentially expressed genes (DEGs) across the exposure conditions (FDR-adjusted *p*-value <0.05). The heatmaps indicate the expression profiles of the top ten genes (sorted first by the number of significant conditions and then by the mean of the absolute fold-changes). The log_2_(fold-changes) compared with the respective air control groups are color-coded and the statistical significance level is indicated (FDR-adjusted *p*-value). (B) Gene set analysis (GSA) was performed with the KEGG gene-set collection using absolute log_2_(fold-changes) as the gene-level and the mean as the gene-set level statistics. Significance with respect to the treatment effect (Q2, compared with the air control) and dominant effects of individual gene sets (Q1) was assessed with Benjamini-Hochberg based FDR adjustment (FDR adj. *p*-value <0.05). The numbers of significantly up- and down-regulated gene sets for Q1 and Q2 are shown in the top panel, and the top gene sets, first sorted by the number of significant conditions and then by their average absolute scores, are shown in the bottom panels. (C) The causal network enrichment approach for the analysis of the transcriptomic datasets. For each network category, the relative biological impact factor is shown in radar plots (CFA, Cell Fate; CPR, Cell Proliferation; CST, Cell Stress; IPN, Inflammatory Process Networks). The heatmaps show the network perturbation amplitudes for each network in the collection, across all conditions. Full details of the comparative analyses for all doses of the exposure are given in the ESI Fig. 2.†

The transcriptomic datasets were used to further compare the exposure-induced perturbation at the cellular and molecular levels. For each organotypic culture, this analysis was done using the data obtained from the cultures exposed to THS2.2 aerosol and 3R4F CS at comparable nicotine concentrations (see Fig. 2, red circles). Doses that elicit limited cellular damage (sub-toxic) were selected to enable evaluation of specific toxicity-related mechanisms associated with exposure while avoiding those merely reflecting severe morphological damage,^52^*e.g.*, those following exposure to high 3R4F CS concentrations. The chosen doses for the comparative evaluation were the following: (1) 3R4F CS at 0.51 mg nicotine per L was compared with THS2.2 aerosol at 0.46 mg nicotine per L in the buccal cultures; (2) 3R4F CS at 0.13 mg nicotine per L was compared with THS2.2 aerosol at 0.14 mg nicotine per L in the bronchial cultures; and (3) 3R4F CS at 0.15 mg nicotine per L was compared with THS2.2 aerosol at 0.15 mg nicotine per L in the nasal cultures. A comparative evaluation across the three epithelium cultures was conducted at the levels of individual genes, gene sets, and causal networks (Fig. 4).

All three organotypic cultures (buccal, bronchial, and nasal) demonstrated a pronounced differential gene expression response upon 3R4F CS exposure at all post-exposure time points (Fig. 4A, top panels). The largest number of differentially expressed genes (DEGs) was observed at 72 h post-exposure to 3R4F CS in the buccal cultures; the largest number of DEGs in the bronchial cultures was observed at 24 h post-exposure following 3R4F CS; whereas the maximum number of DEGs was observed at 4 h post-exposure to 3R4F CS in the nasal cultures. These data suggest a different kinetics of the exposure response across the three cultures. At the similar nicotine concentrations, THS2.2 aerosol elicited a less pronounced change in the DEGs. The maximum number of DEGs was observed 4 h post-exposure to THS2.2 (551 DEGs) in the nasal cultures. In all three cultures, the THS2.2 aerosol-induced DEGs were mostly limited to the earliest post-exposure time point (4 h), suggesting a lower and more transient gene expression response to THS2.2 aerosol exposure than 3R4F CS exposure.

The top ten genes—ranked first by the number of significant conditions and second by their fold-changes—were all involved in either xenobiotic metabolism (CYP1A1, CYP1B1) or the oxidative stress response (TXNRD1, SRXN1, NQO1, HMOX1, SLC7A11, GCLM, LOC344887, MAFF) (Fig. 4A, bottom panels). While 3R4F CS induced persistent changes in these genes at all post-exposure time points, THS2.2 aerosol exposure elicited only transient changes in the expression of these genes, mostly limited to the 4 h post-exposure time point.

Gene-set analysis (GSA) enables aggregation of individual gene responses and links them to specific biological processes. Fig. 4B (upper panels) summarizes the number of significantly affected gene sets of the KEGG gene-set collection.^42^Fig. 4B (lower panels) shows the profiles for the top 10 affected gene sets. Overall, the GSA-based analysis showed a response pattern similar to that of the DEG profiles: 3R4F CS exposure elicited prominent biological responses across all three cultures at all post-exposure times; in contrast, THS2.2 aerosol resulted in a more transient and weaker response (mostly at 4 h post-exposure time point). The large number of diverse gene sets linked to the 3R4F CS exposure suggests an extensive biological impact of 3R4F CS (the entire GSA results are reported in ESI Fig. 1†). The most consistently and most sturdily affected gene sets across the exposure conditions pointed to the activation of xenobiotic (Metabolism of Xenobiotics by Cytochrome P450), oxidative stress (Glutathione Metabolism), and pro-inflammatory (NF-kappa B Signaling Pathway) responses of the exposed cultures. The top 10 gene sets illustrate the main challenge of GSA, whereby unspecific diverse gene sets are uncovered. Such gene sets often are not directly pertinent to the biological context under investigation. For example, the *Pertussis*, *Legionellosis*, and *Chemical Carcinogenesis* gene sets were also identified in this study. Despite its name, *Chemical Carcinogenesis*, the genes within this gene set encode xenobiotic metabolism enzymes. In principle, these xenobiotic metabolizing enzymes can also contribute to the transformation of pro-carcinogens into carcinogens,^53^ but are not directly indicative of an active carcinogenesis process.

As compared with GSA, the causal network enrichment approach offers a more targeted toxicological assessment, in which the relevant biological context is considered [Fig. 1]. In the present work, the exposure-induced perturbation of 29 causal biological network models was analyzed and compared across the buccal, bronchial, and nasal epithelium cultures (with the exception of the Epithelial Mucus Hypersecretion network model, which was not used for the analysis of the buccal cultures as the stratified epithelium model is not comprised of mucus-secreting cells). The analysis demonstrated a similar 3R4F CS-induced perturbation in all four functional network categories for all the three cultures: Cell Stress (CST), Inflammatory Process Network (IPN), Cell Fate (CFT), and Cell Proliferation (CPR) (Fig. 4C, radar plots). The plots demonstrate a pronounced impact of 3R4F CS exposure on the buccal, bronchial, and nasal cultures: the greatest impact was mostly observed at the earliest post-exposure time point and was declining thereafter. However, the buccal cultures’ inflammatory response (modelled in the IPN) following the highest 3R4F CS-induced perturbation was observed at 72 h post-exposure.

Compared with the impact of 3R4F CS, THS2.2 aerosol exposure elicited a lower impact on these network collections. Because the network models are organized hierarchically, further mechanistic investigations can be done at the level of individual networks (Fig. 4C, heatmap panels). For example, in the Cell Stress category, 3R4F CS exposure perturbed the *Xenobiotic Metabolism Response*, *Oxidative Stress*, and *NFE2L2 Signaling* networks in all three cultures. Although THS2.2 aerosol also induced significant perturbations in these networks, the amplitudes of the impact remained well below those observed upon 3R4F CS exposure. The kinetics of the 3R4F CS-induced impact across the three cultures were different. Nasal and bronchial cultures had the highest perturbation at 4 h after 3R4F CS exposure and the perturbations decreased with the duration of post-exposure. Similarly, the buccal cultures were initially perturbed 4 h after 3R4F CS exposure but the impact was lower than that observed in the bronchial and nasal cultures. Thereafter, the perturbations in the buccal cultures generally persisted until the later post-exposure time points—or even exacerbated—unlike those in the bronchial and nasal cultures, *e.g.*, for the *Epithelial Innate Immune Activation* and *Tissue Damage* networks.

The analyses of the transcriptomic data based on the gene, gene-set, and causal network level yielded consistent exposure response profiles with increasing statistical and biological confidence. Each of these analyses provided different levels of mechanistic insights. The DEG analysis demonstrated a differential gene expression profile in response to exposure, especially to 3R4F CS, and could identify the most common genes differentially altered following exposure. The GSA approach could link the impact of exposure to various biological processes, including the impact on xenobiotic metabolism and oxidative stress, despite including some annotations that are less relevant in the context of the respiratory system. Finally, the causal network enrichment methodology provided a more concise quantification of the biological and functional processes that are pertinent for respiratory physiology. Together, the results showed that THS2.2 aerosol exposure induced a weaker and more transient biological impact than 3R4F CS at similar nicotine concentrations, as previously observed.^18^,^19^,^54^ THS2.2 aerosol exposure resulted mainly in transient adaptation processes, which is consistent with the observation that less than 10% of the toxicant yields in 3R4F CS are present in THS2.2 aerosol.^25^

### Clustering of exposure impact based on the pattern of network perturbations

To further assess and summarize the relationships between the selected comparable exposure groups (Fig. 4), clustering based on the causal network perturbation profiles was conducted. For this, two similarity metrics were used: first, amplitude-based, metric compared the magnitudes of the exposure-induced perturbations across the network collection, and second, shape-based, metric specifically considered how the perturbations were distributed relative to each other within individual networks and within the full network collection (a detailed description is given in the Materials and methods section).

**Fig. 5:**
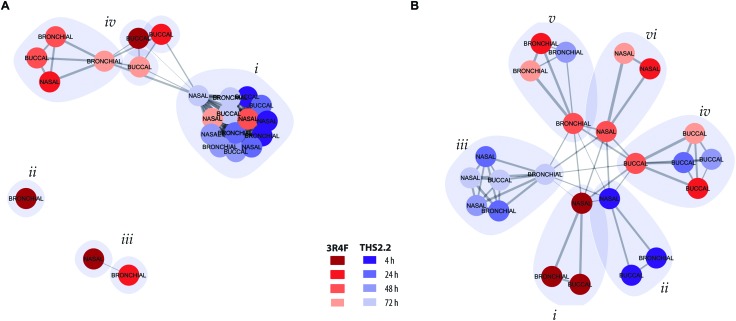
Clustering of exposure conditions based on network perturbations. (A) Clustering of exposure conditions based on the amplitudes of the causal network entities. (B) Clustering based on the shapes of the causal network perturbation profiles without taking the amplitude differences into account. The exposure conditions – compared with their respective sham groups – are represented as colored nodes with the respective culture model as the labels. The post-exposure time points are marked in shades of red for 3R4F CS and in shades of blue for THS2.2 aerosol (see color key). The width of the edges connecting the nodes is proportional to their similarity and identified clusters are demarked with grey polygons.

Clustering with the amplitude-based metric clearly reflected the magnitude of the exposure impact for different exposure groups (Fig. 5A). All THS2.2 exposure impact (at all post-exposure time points for all culture types) were clustered together (Fig. 5A, *i*). However, this cluster also included the later impact of 3R4F CS aerosol exposure on the nasal cultures (48 h and 72 h post-exposure). This observation reflects the overall low and transient network perturbations following THS2.2 aerosol exposure and the rapid recovery of the nasal cultures from the 3R4F CS exposure discussed earlier (*i.e.*, regarding ciliary beating functionality measures (Fig. 3C) and the causal network enrichment analysis (Fig. 4C, radar plots)). The 3R4F CS-induced perturbations at 4 h post-exposure in the bronchial cultures formed an independent cluster (Fig. 5A, *ii*) and those in the nasal cultures and the 3R4F CS-induced perturbations in the bronchial cultures at 24 h post-exposure formed two interconnected clusters (Fig. 5A, *iii*). These findings suggest that the degree of impact on the nasal cultures 4 h after 3R4F CS exposure resembles that on the bronchial cultures at 24 h post-exposure, which was consistent with Fig. 4C showing the network perturbation score. Finally, another cluster (Fig. 5A, *iv*) included the rest of the 3R4F CS-induced impact on the bronchial and nasal cultures at the later post-exposure time points and the 3R4F CS-induced impact on the buccal cultures at all post-exposure time points. This observation resembles the perturbation scores elicited by 3R4F CS exposure shown in Fig. 4C where less impact of exposure in general was observed for the buccal cultures compared with the bronchial and nasal cultures. The overall analysis demonstrated a tissue type specific response following exposure.

To further resolve the similarities of the exposure-induced impact, a cluster analysis was conducted based on the shaped-based metric that relies on the similar pattern of the impacted molecular entities (*i.e.*, network nodes) in the network models, independent of the magnitude (a detailed description is given in the Materials and methods section). If the exposure-induced network perturbations cluster together in this analysis, similar molecular entities were perturbed for the same network model. In contrast, if the exposure impacts do not cluster together, different molecular entities were impacted following exposure, thus the perturbation impacted different network models. Fig. 5B, *i* shows that the perturbations at 4 h after exposure to 3R4F CS for all the three cultures pointed toward similar perturbed molecular entities and network models. Similarly, the THS2.2 aerosol induced impacts at 4 h post-exposure on all culture types formed one cluster (Fig. 5B, *ii*). The THS2.2 aerosol induced impacts at the later post-exposure time points clustered together, suggesting that the transient impact of THS2.2 aerosol exposure affecting similar molecular entities and network models. Furthermore, the shaped-based analysis in particular demonstrated tissue-specific clusters for the exposure impact at the later post-exposure time points: those of buccal (Fig. 5B, *iv*), bronchial (Fig. 5B, *v*), and nasal (Fig. 5B, *vi*).

In summary, the clustering analysis based on the exposure-induced network perturbations enabled a high-level (but mechanism focused) meta-analysis of the exposure responses and could distinguish the lower impact observed following THS2.2 aerosol exposure compared with the more pronounced impact of 3R4F CS (using the amplitude-based metric). In addition, the analysis could identify the similarities and differences in the cellular response across different tissues (especially, using the shape-based metric).

### The impact of exposure on the xenobiotic metabolism and oxidative stress responses

**Fig. 6:**
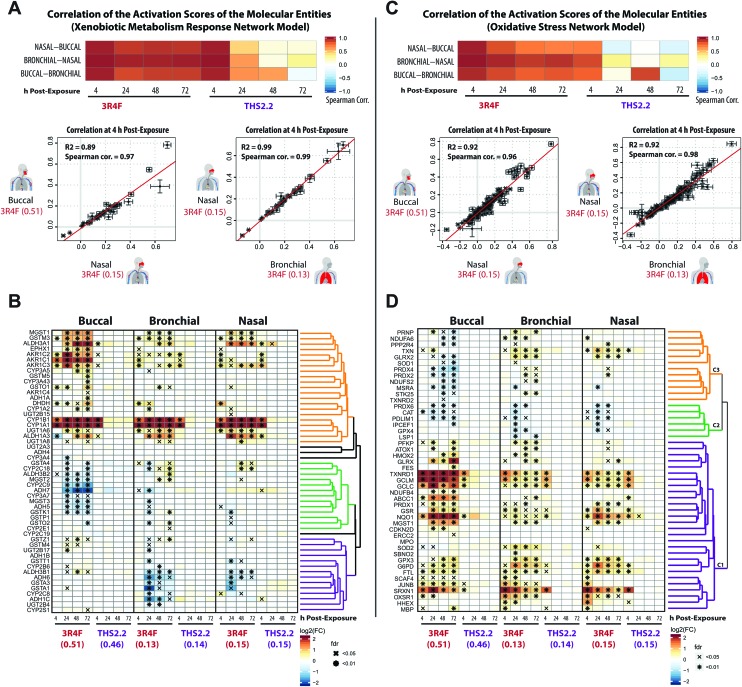
Induction of xenobiotic metabolism and oxidative stress responses across the three organotypic epithelium cultures. (A) Correlation of the perturbation scores of the molecular entities in the Xenobiotic Metabolism Response network across the three organotypic cultures. The color of the heatmap indicates the Spearman correlation coefficients. Scatter plots for each culture pair show the correlation of the activation values of the molecular entities in the Xenobiotic Metabolism Response network. (B) Clustered gene expression matrix for genes of a xenobiotic metabolism gene set (KEGG collection,^42^ Metabolism of Xenobiotics by Cytochrome P450). The log_2_ fold-changes compared with the air control are color-coded and FDR-adjusted significance is indicated for the FDR-adjusted *p*-value <0.05 (*x*) and <0.01 (*) levels. Gene clustering based on the pair-wise correlation between the fold-changes and the clustering results are shown as a dendrogram (clusters are marked in different colors). (C) As in A, but for the Oxidative Stress network. (D) As in B, but for the oxidative stress response gene set (Reactive Oxygen Species Pathway of the hallmark collection^60^).

Some of the most direct cellular changes following CS exposure are the induction of compensatory xenobiotic metabolism and oxidative stress responses.^55^Fig. 4 shows that these response processes were common across the buccal, bronchial, and nasal cultures following 3R4F CS exposure. To further investigate the similarities between these reactions, the correlation of the perturbation scores of the molecular entities (see Fig. 1, panel D) in the Xenobiotic Metabolism Response and Oxidative Stress networks was assessed (Fig. 6A and C, respectively). We further compared the gene expression profiles for the corresponding gene sets of the xenobiotic metabolism response and oxidative stress (Fig. 6B and D).

The correlation plots revealed that the xenobiotic responses to 3R4F CS were well aligned across the three cultures. The perturbation scores of the molecular entities within the Xenobiotic Metabolism Network exhibited high Spearman correlation values across the buccal, bronchial, and nasal cultures (Fig. 6A). The highest correlation values were observed for the 4 h time points following 3R4F CS exposure. Because of the minor (and mainly transient) biological impact of THS2.2 aerosol exposure, the correlation values for the cultures exposed to THS2.2 aerosol were well correlated only at the 4 h post-exposure time point. This is consistent with the observed transient xenobiotic response following THS2.2 aerosol exposure shown in Fig. 4. Furthermore, the expression profiles of genes involved in xenobiotic metabolism (Fig. 6B) showed that genes encoding the cytochrome P450 enzymes CYP1A1 and CYP1B1 were greatly induced in response to 3R4F CS exposure. This observation was consistent in all three organotypic cultures. Moreover, the increased CYP1A1 and CYP1B1 levels only at 4 h post-exposure to THS2.2 aerosol further exemplified the earlier remark regarding the transient response upon exposure to THS2.2 aerosol. Additional xenobiotic metabolism genes—with a similar (transiently altered) profile—were assigned to the orange correlation cluster (*i.e.*, aldehyde dehydrogenases ALDH1A3 and ALDH3A1, aldo-keto reductases such as AKR1C3, and the glucuronosyltransferase UGT1A6). Notably, the expression profiles of some genes in this orange cluster suggested differential xenobiotic responses between the buccal (stratified epithelium) and bronchial/nasal (pseudostratified epithelium) cultures; for example, the epoxide hydrolase EPHX1 gene, which was most prominently up-regulated in the buccal culture following 3R4F CS exposure. The genes within the green cluster were mostly down-regulated in the buccal as compared with the bronchial/nasal cultures; whereas the genes within the purple cluster showed the opposite trend. This analysis further showed that several paralogous genes were assigned to different clusters (*e.g.* for the green *vs.* purple cluster: GSTA4 *vs.* GSTA1/3, ADH5/7 *vs.* ADH1B/6, ADH3B2 *vs.* ADH3B1, and CYP2C9 *vs.* CYP2C8 were assigned). These results suggest tissue-specific differences in the xenobiotic metabolism response, which involve different members of these gene families.

The correlations of the perturbation scores of the molecular entities within the Oxidative Stress network showed a fairly consistent response across the three cultures following 3R4F CS exposure at all post-exposure time points. For THS2.2 aerosol exposure, high correlation values were only observed for the 4 h post-exposure time points (Fig. 6C). A correlation-based clustering of the expression profiles of oxidative stress-responsive genes revealed three main clusters (Fig. 6D): the genes within the main cluster (purple) showed a consistent induction after 3R4F CS exposure for buccal, bronchial, and nasal cultures. This cluster contains genes that represent the main branches of the oxidative stress response, namely: the glutathione system (GCLC, GCLM, MGST1, GSR), the thioredoxin system (thioredoxin reductase 1, TXNRD1), the peroxiredoxin system (PRDX1, SRXN1), quinone NAD(P)H dehydrogenase 1 (NQO1), and metabolic adjustments for NAPDH production (glucose-6-phosphate dehydrogenase (G6PD)). A similar impact on the oxidative stress response pathways was observed previously in rat lung tissue exposed to 3R4F CS^56^ and in human lung tissue under oxidative stress conditions.^57^–^59^ The altered gene expression in this cluster further showed that THS2.2 aerosol induced a transient oxidative stress response that was limited to the 4 h after exposure time point across all three cultures (an exception was observed for NQO1 in the nasal culture). The second cluster (green) contains several genes including the gene encoding catalase (CAT) that were significantly down-regulated only upon 3R4F CS exposure in all three cultures. This is different from a previous study, which found that CS exposure up-regulated the expression of CAT.^59^ Moreover, following repeated exposure to 3R4F CS, the expression of the CAT gene in human organotypic gingival cultures was found to be up-regulated.^54^ The third cluster of genes (orange) indicates a distinct oxidative stress response. Genes in this cluster were mainly down-regulated in the buccal (stratified epithelium) but mainly up-regulated in both bronchial and nasal (pseudostratified epithelium) cultures in response to 3R4F CS exposure.

Taken together, both 3R4F CS and THS2.2 aerosol exposure affected the xenobiotic metabolism and oxidative stress response of the three cultures. Both processes were consistently perturbed following 3R4F CS exposure across all three tissue cultures; however THS2.2 aerosol exposure elicited a weak and mostly transient response (occurring only at the early 4 h post-exposure time point). The results also suggest that some specific gene isoforms that are involved in both the xenobiotic and oxidative responses were induced in a tissue-specific manner following the exposures. Such information could identify a distinct response profile across the buccal, bronchial, and nasal epithelium cultures.

### The impact of exposure on the inflammatory responses

Cigarette smoking modulates inflammation and promotes chronic inflammation. CS is known to impact host immunity, including the innate immunity in the oral, nasal, and airway mucosa that eventually affects the adaptive immunity at the systemic level.^61^ In this work, a further investigation of the tissues’ inflammatory responses following exposure was performed based on the transcriptomic datasets and secreted pro-inflammatory mediator data collected at various post-exposure time points.

**Fig. 7:**
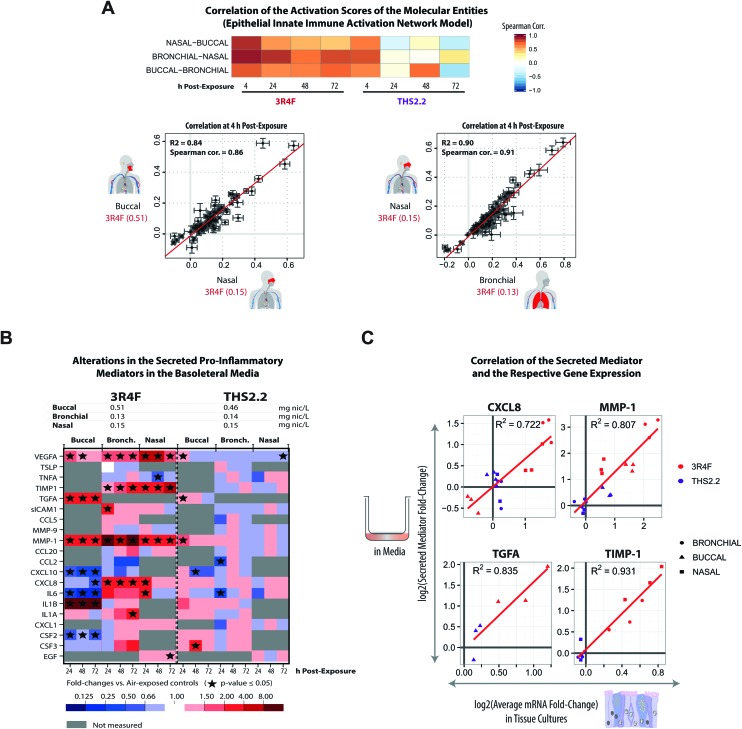
Exposure-induced pro-inflammatory responses across the buccal, bronchial, and nasal cultures. (A) Correlations of the perturbation scores of the molecular entities in the Epithelial Innate Immune Activation network across the three organotypic models. (B) Multianalyte profiling data for secreted pro-inflammatory mediators measured at various post-exposure time points (as a cross-sectional sampling) are shown. The fold-changes relative to the respective sham groups are color coded. Grey cells indicate that no measurement was conducted for the specific mediators. ESI Fig. 3† shows the data for all the tested concentrations. (C) Correlations between the secreted mediators and their corresponding mRNAs (based on the transcriptomic data) for CXCL8, MMP1, TGFA, and TIMP-1 are shown. Correlation plots for the complete set of mediators and concentrations are reported in ESI Fig. 4.† The log_2_ fold-change (relative to the air control) of a secreted mediator at a given post-exposure time point (*i.e.* 24 h, 48 h, and 72 h) (*y*-axis) is compared with the average log_2_ fold-change (relative to the air control) of the respective mRNA for the same and preceding times to account for accumulation of the secreted protein products (*x*-axis).

Based on the causal network enrichment analysis of the transcriptomic data, Fig. 4 shows that for the three organotypic epithelium cultures, 3R4F CS exposure elicited a common inflammatory response mechanism (as modeled in the Epithelial Innate Immune Activation network). Furthermore, a correlation analysis was conducted, for which the perturbation scores for the molecular entities in this network (see Fig. 1, panel D) were correlated across the three organotypic cultures. Fig. 7A showed that 4 h post-exposure to 3R4F CS, the perturbation scores of the molecular entities of the Epithelial Innate Immune Activation network, across the three tissues, were highly correlated. Higher Spearman correlations were observed for the comparison between the bronchial and nasal cultures (both of which are pseudostratified epithelium) as compared with the correlations between the bronchial/nasal and the stratified buccal epithelium cultures (despite a fairly high correlation at the 4 h post-exposure time point, Fig. 7A). The lower correlation between bronchial/nasal and buccal may reflect the previously reported differences in the immune response between the buccal (stratified epithelium) and bronchial/nasal (pseudostratified epithelium) cultures. For example, differences in the induction of antimicrobial human beta-defensins (hBDs) have been reported: hBDs are induced by TNFα, IL-1, and phorbol 12-myristate 13-acetate, but not by lipopolysaccharides in the oral epithelium; whereas lipopolysaccharides induced the gene expression of hBDs in the tracheal epithelium.^62^ Another possible reason for this discrepancy could be attributed to the thicker morphology of the buccal cultures, or to the different donors from which the primary cells were isolated for the generation of the culture models. The stratified epithelium may trigger a delayed kinetic response to exposure (discussed earlier). Furthermore, as shown in Fig. 4, THS2.2 aerosol exposure elicited only small alterations in the global gene expression of the buccal, bronchial, and nasal cultures, except at the 4 h post-exposure time point. The perturbation scores of the molecular entities within the Epithelial Innate Immune Activation network model were much lower as compared with that for 3R4F CS exposure (Fig. 4). As a result, the correlation values between the perturbation scores of the molecular entities within the Epithelial Innate Immune Activation were fairly low across the three cultures (and only well correlated at the 4 h post-exposure time point, Fig. 7A). This analysis demonstrates that the causal-network enrichment approach could further identify the exposure-related molecular mechanisms that are common (and different) across the three epithelium cultures.

For the analyzed panel of secreted pro-inflammatory mediators, 3R4F CS exposure resulted in shared but also distinct effects across the three culture models – with a higher similarity between bronchial and nasal than buccal cultures (Fig. 7B, ESI Fig. 3†). A generally consistent increase in matrix metalloproteinase 1 (MMP-1) levels was observed at all post-exposure time points following 3R4F CS. The association between CS and the increased expression of MMP-1 has been reported previously.^63^ In lung epithelial cells, the link between CS and emphysema has been attributed to the alteration in MMP-1 levels.^63^ Increased levels of the secreted vascular endothelial growth factor alpha (VEGFA) were also observed following 3R4F CS exposure in all three culture models. In contrast, CXCL8 and IL6 levels were decreased following 3R4F CS in the buccal culture, but tended to be increased in the bronchial and nasal cultures. This observation further supports the distinct inflammatory response between the pseudostratified epithelium (bronchial/nasal) and stratified epithelium (buccal) discussed earlier. The effects of THS2.2 aerosol exposure on these pro-inflammatory mediators generally remained lower and less commonly significant than those upon 3R4F CS exposure at these comparable doses. Finally, an integrative analysis of RNA and protein levels revealed good correlations for CXCL8, MMP-1, TGFA, and TIMP-1 levels (Fig. 7C), suggesting that these mediators are regulated predominantly at the transcriptional level.

### Alterations of microRNAs following exposure

**Fig. 8:**
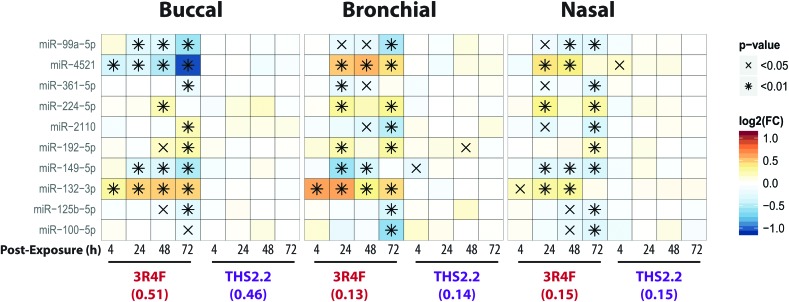
Differentially expressed microRNAs following exposure in the buccal, bronchial, and nasal cultures. The heatmap shows miRNAs with significant differential expression in at least one comparison in all three epithelium cultures. Each row represents one microRNA, each column represents a comparison between one exposed sample *versus* its air control, with log_2_(fold-changes) color-coded and significance levels indicated.

MicroRNAs (miRNAs) are involved in the post-transcriptional regulation of diverse biological processes.^64^ To identify the miRNA-based regulation following exposure that is commonly observed in the buccal, bronchial, and nasal cultures, miRNAs that were found to be significantly differentially expressed in at least one comparison were evaluated (Fig. 8).

Across the three cultures, 3R4F CS exposure was linked to a reduced expression of miR-99a, miR-361, miR-149, miR-125b, and miR-100. Various studies have demonstrated the role of miR-99a in growth factor signaling pathways, including the insulin-like growth factor 1 receptor (IGF-1R) and mammalian/mechanistic target of rapamycin (mTOR).^65^–^68^ The miR-99a is thought to regulate cell proliferation and differentiation, although many studies were done using cancer cells.^66^–^70^ In contrast, the levels of miR-99a in the cultures exposed to THS2.2 aerosol were not significantly different from the air control.

Increased levels of miR-224, miR-192, and miR-132 following 3R4F CS exposure were consistently found in all epithelium cultures (buccal, bronchial, and nasal). A study has shown that miR-132 regulates cell proliferation by suppressing the retinoblastoma protein (RB1).^71^ Following allergen exposure, the levels of miR-132 in the bronchial brushing samples were increased, which was linked to shedding of bronchial epithelial cells *via* the repression of cyclin dependent kinase inhibitor 1A (CDKN1A).^72^ Moreover, following exposure to ozone, increased miR-132 expression in human sputum cells was reported.^73^ The levels of miR-132 were not impacted by the THS2.2 aerosol exposure at all post-exposure time points. The levels of miR-4521 were regulated differently following exposure to 3R4F CS: decreased expression was observed in the buccal cultures but increased expression was observed in the bronchial and nasal cultures. A study has reported that the single nucleotide polymorphism rs7210250—located near miR-4521 loci—was associated with risk of esophageal adenocarcinoma.^74^ Tobacco smoking is one of the risk factors of the disease. The observed difference in the directionality of the miR-4521 expression following 3R4F CS across the cultures could be attributed to the different tissue type or the different donor profile from which the organotypic cultures were reconstituted. Nevertheless, the specific role of miR-4521 has not been reported.

Overall, eight out of the ten miRNAs, which were significantly differently expressed compared with the air controls, were similarly altered following 3R4F CS and THS2.2 aerosol exposure in all three cultures (Fig. 8), demonstrating a common regulatory miRNA-profile across the buccal, bronchial, and nasal cultures. Consistent with the other endpoints analyzed in this study, THS2.2 aerosol exposure in general elicited reduced impact on the alterations in miRNAs compared with that following 3R4F CS exposure at comparable nicotine concentrations.

### A targeted proteomics analysis: exposure-induced cellular stress in the nasal cultures

An additional data modality, a protein marker panel using targeted proteomics, was evaluated for the nasal culture samples collected 48 h after exposure. Such data are aimed at increasing the robustness of a toxicity assessment. The additional data modality will further support the observed biological effects based on the other functional measurements, such as the multi-analyte profile of the secreted pro-inflammatory mediators and transcriptomic data.

**Fig. 9:**
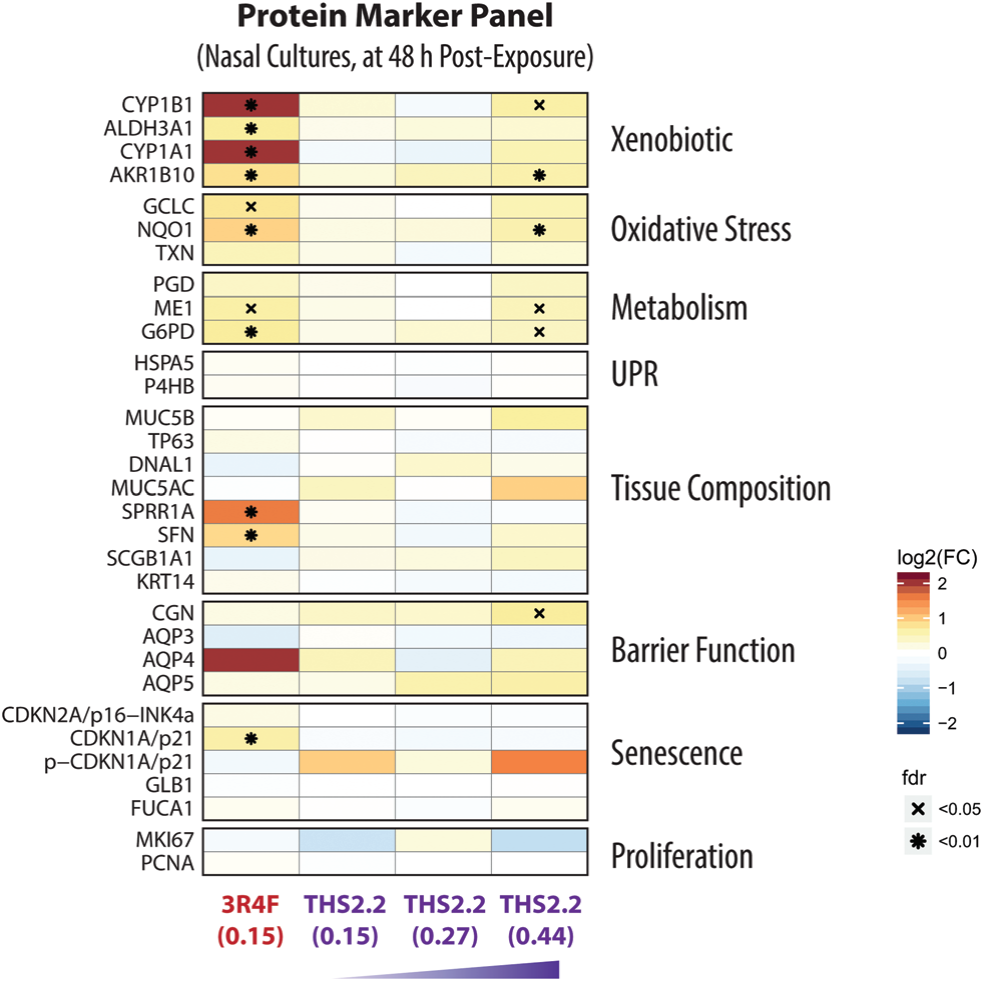
Alterations of proteins in the nasal organotypic cultures following exposure. A panel of marker proteins was quantified by targeted mass-spectrometry based proteomics for the 48 h post-exposure time point for the nasal culture. Each row represents one quantified protein, and each column represents a comparison between an exposed sample *versus* its air control. The log_2_(fold-changes) compared with its air control are color-coded and the FDR-adjusted *p*-values are indicated. Protein marker categories are indicated on the left side of the heatmap (UPR, unfolded protein response).

Fig. 9 shows the expression of various proteins that were altered at 48 h after exposure measured using mass spectrometry-based targeted proteomics. Exposure to 3R4F CS was associated with increased levels of proteins involved in the xenobiotic protein response. First, the cytochrome P450 enzymes CYP1A1 and CYP1B1 had the strongest up-regulation following 3R4F CS exposure. Both CYP1A1 and CYP1B1 are known to be regulated upon activation of the arylhydrocarbon receptor following exposure to xenobiotics.^75^ Second, the levels of the aldehyde dehydrogenase 3A1 (ALDH3A1) protein were increased in the nasal cultures exposed to 3R4F CS compared with the air controls. ALDH3A1 is frequently found to be up-regulated following CS exposure; for example, increased levels of ALDH3A1 were detected in the fluid lining the epithelial cells in smokers.^76^ Moreover, the abundance of ALDH3A1 in the sputum could distinguish smokers from non-smokers, and thus can serve as a specific marker of the smoking status.^77^ These observations demonstrate consistently the greater levels of ALDH3A1 after CS exposure both *in vivo* and *in vitro*. Finally, the increased expression of AKR1B10, an aldo-keto-reductase, following 3R4F CS exemplifies the culture response against toxic aldehydes and CS exposure, as reported previously.^78^,^79^ Overall, the alteration in the protein markers further confirmed that 3R4F CS exposure impacted the xenobiotic metabolism response of the nasal cultures (based on the network enrichment analysis of the transcriptomic data).

Furthermore, the proteomics data also support the 3R4F CS-induced oxidative stress discussed earlier. The protein levels of both the catalytic subunit of the glutamate cysteine ligase (GCLC) and the NAD(P)H-quinone dehydrogenase 1 (NQO1) were significantly increased in the nasal cultures exposed to 3R4F CS compared with the air control (Fig. 7). GCLC is the catalytic subunit of the rate limiting enzyme for glutathione synthesis; thus GCLC is essential for the oxidative stress reaction (which ensues following smoke exposure).^58^ NQO1 is known as an oxidative stress enzyme, and acts as a quinone reductase. Increased levels of NQO1 following CS exposure in the sputum of smokers has been reported previously.^77^ Similarly, increased NQO1 levels were observed in the large airway epithelium of smokers.^80^ In addition, the generation of NADPH is essential for the cellular response against oxidative stress.^59^,^81^,^82^ In this study, the two key enzymes regulating the generation of NADPH—glucose-6-phosphate dehydrogenase (G6PD) and malate enzyme (ME1)—were up-regulated following 3R4F CS exposure. Overall, the proteomics data further confirm that 3R4F CS induced oxidative stress response in the nasal cultures.

These proteomics data could further confirm the findings obtained using the causal network enrichment approach. For example, based on the transcriptomic data, 3R4F CS exposure was linked to a significant perturbation of the *Senescence* network model (Fig. 4C). Here, the proteomics analysis identified that the expression of p21—a cell cycle regulator known to accumulate in senescent cells^83^,^84^—was increased following 3R4F CS (Fig. 9). In addition, the increased expression of stratifin (SFN) and the small proline rich protein 1A (SPRR1A) following 3R4F CS could be linked to the significant perturbation of the Tissue Damage network (Fig. 4C). The expression of SFN is mainly associated with squamous epithelial cells,^85^ thus suggesting that 3R4F CS exposure might trigger a cellular differentiation in the nasal cultures after exposure. Moreover, SPRR1A plays a role in the barrier function of the epithelium.^86^ Another isoform of the protein, SPRR3, was also reported to be increased in the sputum and large airway epithelium of smokers.^77^,^80^

Consistent with the other data modalities (functional measures, secreted mediator profiles, and transcriptomics), when compared to the impact of 3R4F CS, THS2.2 aerosol exposure resulted in much more limited alterations of protein expression. Only when the nasal cultures were exposed to THS2.2 aerosol at a nicotine concentration three times that of 3R4F CS, increased levels of a subset of the proteins were observed (CYP1B1, AKR1B10, NQO1, ME1, G6PD, and CGN) (Fig. 9). Nevertheless, the degree to which these proteins were altered remained lower than that observed following 3R4F CS exposure. Overall, the results illustrate that an additional data modality—in this present study, the proteomics data—can further support and confirm the observations based on the analysis of transcriptomic data *i.e.*, the reduced impact of THS2.2 aerosol as compared with 3R4F CS was linked to the reduced xenobiotic metabolism and oxidative stress responses.

## Conclusions and outlook

According to the “Toxicity Testing in the 21st Century: a Vision and a Strategy” published by the U.S. National Academy of Sciences in 2007, tremendous improvement in toxicological assessment can be accomplished by leveraging high-throughput measurements together with biological models and systems biology approaches.^5^,^87^ The Vision and Strategy promotes the use of more relevant *in vitro* models for human physiology, which minimizes animal testing. Several projects have subsequently applied the strategy, such as the ToxCast and Tox21 programs.^88^,^89^ Similarly, our group has developed a five-step systems toxicology approach [Fig. 1] that relies on causal network models to enable a mechanism-based quantification of an exposure impact based on omics data in a relevant biological context. In the current work, we demonstrate the applicability of this approach not only to quantify but also to compare the effects of 3R4F CS and THS2.2 aerosol exposure at the level of pertinent biological mechanisms across three organotypic culture models. This approach allowed a comprehensive comparative assessment of the biological and toxicological responses of tissues lining the aerodigestive tract from the oral cavity, the lung, and the nasal cavity (the “field of injury”) following exposure to 3R4F CS and THS2.2 aerosol. This work further indicated that the 21^st^ century toxicology approach can be performed effectively in the 3Rs context: to reduce, refine, and/or replace animal testing.^8^

The present meta-analysis includes functional measurements (cytotoxicity, ciliary beating functionality, and pro-inflammatory mediator profiles) and advanced computational approaches leveraging gene set analyses and causal network enrichment to comprehensively assess the biological impact of 3R4F CS and THS2.2 aerosol exposures on the *in vitro* human organotypic buccal, bronchial, and nasal cultures. An exposure characterization was included in the assessment to ensure the reproducibility and the consistency of the smoke/aerosol generation throughout the studies. On the mechanistic level, the responses of the three organotypic cultures to 3R4F CS were well aligned, with a prominent engagement of xenobiotic metabolism, oxidative stress, and pro-inflammatory mechanisms. Buccal tissues demonstrated an overall lower sensitivity to the cytotoxic effects of 3R4F CS exposure, which could be attributed to the thicker layer of the buccal culture (a stratified epithelium) and the different donors from which the primary cells were isolated (Fig. 2). The culture responses—based on the transcriptome changes—could further demonstrate that THS2.2 aerosol exposure elicited a more transient response (at the 4 h post-exposure time point) in all the three cultures. The analysis further demonstrated some differences, for example, that specific isoforms of genes or proteins were regulated differently across the three cultures following exposure. The analysis based on the various data modalities further confirmed the overall reduced and more transient biological impact of THS2.2 aerosol exposure, as compared with 3R4F CS. The similarity of study design across the three studies (buccal, bronchial, and nasal) enabled a robust and reproducible analysis, which supported an overall reduced impact of THS2.2 aerosol exposure on the “field of injury” tissues *in vitro*, compared with that of CS.

In the current studies, these *in vitro* models were used for the comparative assessment of smoke/aerosol at similar nicotine concentrations, to investigate relative differences in the effects elicited in biologically relevant culture systems. Currently, the *in vitro* exposure scenario does not directly mimic the exposure situation in the aerodigestive tract – *e.g.*, because of differences in the puffing parameters, the thermodynamic state of the flowing smoke/aerosol, and the geometry of the flow path *in vitro* and *in vivo*. Therefore, we have ongoing efforts in our group^90^,^91^ that incorporate computational fluid dynamics models to further refine our knowledge and understanding of smoke/aerosol behavior in the human respiratory tract and *in vitro* systems – to eventually allow for more direct translatability between the culture systems and the *in vivo* situation.

Overall, the present paper demonstrates that the systems toxicology approach is a robust methodology that can not only detect toxic effects of exposure, but also infer the mechanistic-based toxicity assessment. Moreover, this approach could deduce relevant biological mechanisms, such as cell stress and inflammatory processes—which are relevant for the pathophysiological effects of smoking in humans—thus offering an opportunity for translation to tissue-specific clinical biomarkers.

This work also exemplifies how targeted proteomics can strengthen the findings observed using the causal network enrichment analysis based on transcriptomic data: the xenobiotic metabolism and oxidative stress responses were prominently elicited by 3R4F CS and only slightly impacted by THS2.2 aerosol exposure. In the future, the systems toxicology approach will mature as additional omics data modalities will be incorporated. Such multimodality data will enable investigations into the exposure effects at multiple levels of biological organization. In previous work, our group and others have demonstrated that multi-omics profiling can unravel toxicological mechanisms: an integration of transcriptomics, proteomics, and metabolomics resulted in a comprehensive characterization of the effects of Cyclosporine A and cisplatin on cell stress in renal epithelial cells;^92^,^93^ a combination of transcriptomics and metabolomics revealed that reduced oxidative stress was linked to THS2.2 aerosol exposure as compared with exposure to CS in human organotypic gingival cultures;^54^ and integration of transcriptomics, proteomics, and lipidomics data revealed the impact of candidate MRTP aerosols and CS on lipid metabolism in mouse lungs.^82^ In the future, it will be pertinent to directly integrate proteomics results with the causal network approach, leading to the generation of multi-scale network models.

The scientific community will play an important role in the success of systems toxicology as an assessment approach. Indeed, making the network models, algorithms, and datasets available to the scientific community is critical to validate the applied methodologies. Motivated by the Tox21 94 and the OpenTox projects,^95^ which incorporate public repositories of large-scale toxicity data, the Causal Biological Network Database (CBN) has been made available to share the Causal Biological Network Models (; http://www.causalbionet.com/); . 29 The Systems Biology Verification project (sbvIMPROVER) (; http://sbvimprover.com) was organized to evaluate microarray-based phenotype predictions,^96^ species translation,^97^ generation of a comprehensive set of COPD-relevant models,^36^ and recently blood-based gene expression signatures to classify exposure status between 3R4F CS and MRTP aerosols.^98^ Furthermore, the INTERVALS platform has been developed to share results from *in vivo* inhalation studies and *in vitro* studies in the context of product assessment.^99^

**Fig. 10:**
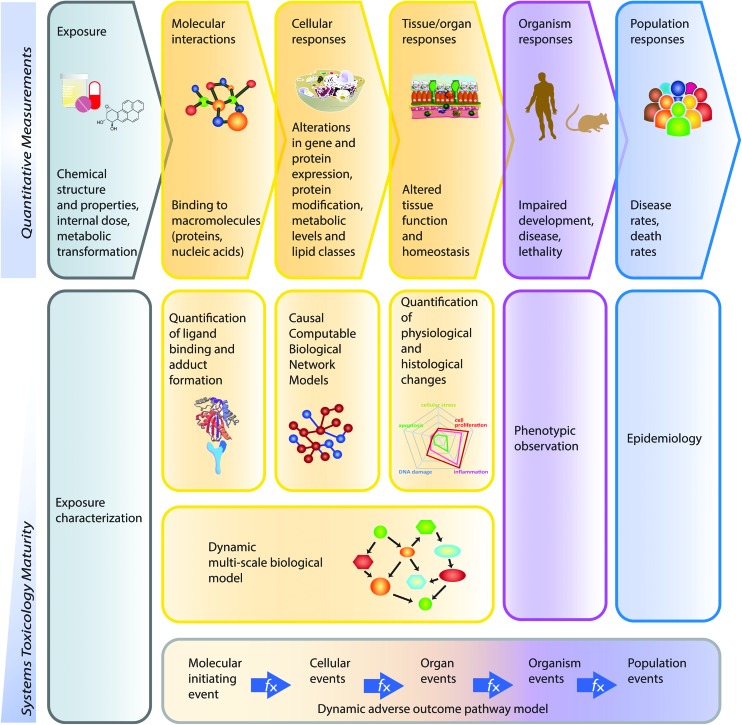
Dimensions of the systems toxicology paradigm. Toxicological effects percolate through biological systems, from the initiating molecular interactions to cellular and tissue responses to population effects (upper panels). Different measurement techniques enable these effects to be quantitatively pursued at all levels. As systems toxicology evolves, additional levels of the exposure effect can be captured in a single mathematical model (lower panel). In this study, the causal network models captured the cellular responses based on transcriptomic measurements, which were complemented by additional functional measurements, *e.g.* cytotoxicity and cilia beating analysis. The development of dynamic multi-scale models will allow a uniform assessment of all levels of the exposure effects from molecular interactions to tissue/organ responses. Eventually, dynamic adverse outcome pathway (AOP) models will bridge the entire toxicological response, from molecular initiating events to population effects. Adapted from the artwork of Samantha J. Elmhurst (; http://www.livingart.org.uk) published previously.^4^

In the future, the systems toxicology approach—guided by the adverse outcome pathway (AOP) framework^100^—is envisioned to mature and eventually advance into dynamic AOPs that can be used to quantify and/or predict all the steps of a toxicological response. Such a response is ranging from the initial molecular interactions between the toxicant and the host system (molecular initiating event), to the cellular /organ events, and finally to the organism- and population-level events^4^ (Fig. 10). An integrative systems toxicology assessment framework can be broadly applied to all relevant areas of toxicology, including food safety, pharmacological drug safety, and occupational safety. Although the present work reports the application of the systems toxicology approach in the context of tobacco product assessment, the same approach has already been shown to be applicable for other toxicity evaluations, such as for nutraceuticals.^101^ A literature curation platform for causal network models has been established that enables a straight forward expansion to other disease areas, as was demonstrated for atherosclerosis plaque destabilization.^102^ Indeed, this framework is envisioned to evolve beyond traditional toxicological assessment approaches. For example, incorporating genetics and other personalized risk factors will allow an individual-based safety assessment, similar to personalized medicine.^103^ Beyond toxicological assessment, this approach also has the potential to generate novel insights into disease mechanisms and support the identification of pharmaceutical interventions, as already proposed for the AOP framework.^100^ Thus, continuing further on this path, the outlined strategy and approaches are well equipped to make the vision of 21^st^ century toxicology a reality.^5^,^87^

The meta-analysis reported here demonstrates the applicability of the systems toxicology approach to generate comprehensive and consistent data showing the culture response to 3R4F CS and THS2.2 aerosol on several relevant biological mechanisms, including cellular stress and pro-inflammatory responses. The results show consistently across all three *in vitro* models—buccal, bronchial, and nasal—that THS2.2 aerosol exposure had a considerably reduced and more transient biological impact on these *in vitro* models compared with equivalent exposures to 3R4F CS.

## Conflict of interest

All authors are employees of or consultants (W.K. Schlage) paid by Philip Morris International. Philip Morris International is the sole source of funding and sponsor of this project.

## Author contributions

ARI, BT, AS, PL, CM, FM, MCP, and JH contributed to the conception or design of the work. ARI, TS, FZ, CM, AE, SF, and NVI contributed to the data collection. ARI, BT, AS, PL, TS, FZ, CM, WS, FM, and JH contributed to the data analysis and interpretation. ARI, BT, and JH drafted the article. All authors critically revised and approved the article.

## Supplementary Material

Supplementary information

## References

[cit1] Fang F. C., Casadevall A. (2011). Infect. Immun..

[cit2] Ideker T., Galitski T., Hood L. (2001). Annu. Rev. Genomics Hum. Genet..

[cit3] Peitsch M. C., de Graaf D. (2014). Drug Discovery Today.

[cit4] Sturla S. J., Boobis A. R., FitzGerald R. E., Hoeng J., Kavlock R. J., Schirmer K., Whelan M., Wilks M. F., Peitsch M. C. (2014). Chem. Res. Toxicol..

[cit5] National Research Council. Committee on Toxicity Testing Assessment of Environmental Agents, Toxicity testing in the 21st century: A vision and a strategy, National Academies Press, 2007.

[cit6] Yick C. Y., von der Thüsen J. H., Bel E. H., Sterk P. J., Kunst P. W. (2011). Respir. Res..

[cit7] Thiberville L., Salaun M., Lachkar S., Dominique S., Moreno-Swirc S., Vever-Bizet C., Bourg-Heckly G. (2009). Eur. Respir. J..

[cit8] Ferdowsian H. R., Beck N. (2011). PLoS One.

[cit9] Grego S., Dougherty E. R., Alexander F. J., Auerbach S. S., Berridge B. R., Bittner M. L., Casey W., Cooley P. C., Dash A., Ferguson S. S., Fennell T. R., Hawkins B. T., Hickey A. J., Kleensang A., Liebman M. N., Martin F., Maull E. A., Paragas J., Qiao G. G., Ramaiahgari S., Sumner S. J., Yoon M. (2016). Altex.

[cit10] Knudsen T. B., Keller D. A., Sander M., Carney E. W., Doerrer N. G., Eaton D. L., Fitzpatrick S. C., Hastings K. L., Mendrick D. L., Tice R. R., Watkins P. B., Whelan M. (2015). Toxicol. Sci..

[cit11] Warnakulasuriya S., Dietrich T., Bornstein M. M., Casals Peidro E., Preshaw P. M., Walter C., Wennstrom J. L., Bergstrom J. (2010). Int. Dent. J..

[cit12] Winn D. M. (2001). J. Dent. Educ..

[cit13] Sasco A. J., Secretan M. B., Straif K. (2004). Lung Cancer.

[cit14] U. S. Office of the Surgeon General, The Health Consequences of Smoking: A Report of the Surgeon General, Centers for Disease Control and Prevention (US), Atlanta (GA), 2004. 20669512

[cit15] Sridhar S., Schembri F., Zeskind J., Shah V., Gustafson A. M., Steiling K., Liu G., Dumas Y. M., Zhang X., Brody J. S., Lenburg M. E., Spira A. (2008). BMC Genomics.

[cit16] Zhang X., Sebastiani P., Liu G., Schembri F., Zhang X., Dumas Y. M., Langer E. M., Alekseyev Y., O'Connor G. T., Brooks D. R., Lenburg M. E., Spira A. (2010). Physiol. Genomics.

[cit17] Steiling K., Ryan J., Brody J. S., Spira A. (2008). Cancer Prev. Res..

[cit18] Iskandar A. R., Mathis C., Martin F., Leroy P., Sewer A., Majeed S., Kuehn D., Trivedi K., Grandolfo D., Cabanski M., Guedj E., Merg C., Frentzel S., Ivanov N. V., Peitsch M. C., Hoeng J. (2016). Altex.

[cit19] Iskandar A. R., Mathis C., Schlage W. K., Frentzel S., Leroy P., Xiang Y., Sewer A., Majeed S., Ortega-Torres L., Johne S., Guedj E., Trivedi K., Kratzer G., Merg C., Elamin A., Martin F., Ivanov N. V., Peitsch M. C., Hoeng J. (2017). Toxicol. In Vitro.

[cit20] Zanetti F., Sewer A., Mathis C., Iskandar A. R., Kostadinova R., Schlage W. K., Leroy P., Majeed S., Guedj E., Trivedi K., Martin F., Elamin A., Merg C., Ivanov N. V., Frentzel S., Peitsch M. C., Hoeng J. (2016). Chem. Res. Toxicol..

[cit21] Hoeng J., Talikka M., Martin F., Sewer A., Yang X., Iskandar A., Schlage W. K., Peitsch M. C. (2014). Drug Discovery Today.

[cit22] Ben-Shlomo S., Zvibel I., Rabinowich L., Goldiner I., Shlomai A., Santo E. M., Halpern Z., Oren R., Fishman S. (2013). Dig. Dis. Sci..

[cit23] Mathis C., Poussin C., Weisensee D., Gebel S., Hengstermann A., Sewer A., Belcastro V., Xiang Y., Ansari S., Wagner S., Hoeng J., Peitsch M. C. (2013). Am. J. Physiol.: Lung Cell. Mol. Physiol..

[cit24] Talikka M., Kostadinova R., Xiang Y., Mathis C., Sewer A., Majeed S., Kuehn D., Frentzel S., Merg C., Geertz M., Martin F., Ivanov N. V., Peitsch M. C., Hoeng J. (2014). Int. J. Toxicol..

[cit25] Schaller J. P., Keller D., Poget L., Pratte P., Kaelin E., McHugh D., Cudazzo G., Smart D., Tricker A. R., Gautier L., Yerly M. (2016). Regul. Toxicol. Pharmacol..

[cit26] Hoeng J., Deehan R., Pratt D., Martin F., Sewer A., Thomson T. M., Drubin D. A., Waters C. A., de Graaf D., Peitsch M. C. (2012). Drug Discovery Today.

[cit27] Martin F., Thomson T. M., Sewer A., Drubin D. A., Mathis C., Weisensee D., Pratt D., Hoeng J., Peitsch M. C. (2012). BMC Syst. Biol..

[cit28] Martin F., Sewer A., Talikka M., Xiang Y., Hoeng J., Peitsch M. C. (2014). BMC Bioinf..

[cit29] Boué S., Talikka M., Westra J. W., Hayes W., Di Fabio A., Park J., Schlage W. K., Sewer A., Fields B., Ansari S. (2015). Database.

[cit30] Westra J. W., Schlage W. K., Frushour B. P., Gebel S., Catlett N. L., Han W., Eddy S. F., Hengstermann A., Matthews A. L., Mathis C. (2011). BMC Syst. Biol..

[cit31] Schlage W. K., Westra J. W., Gebel S., Catlett N. L., Mathis C., Frushour B. P., Hengstermann A., Van Hooser A., Poussin C., Wong B., Lietz M., Park J., Drubin D., Veljkovic E., Peitsch M. C., Hoeng J., Deehan R. (2011). BMC Syst. Biol..

[cit32] Gebel S., Lichtner R. B., Frushour B., Schlage W. K., Hoang V., Talikka M., Hengstermann A., Mathis C., Veljkovic E., Peck M. (2013). Bioinf. Biol. Insights.

[cit33] Westra J. W., Schlage W. K., Hengstermann A., Gebel S., Mathis C., Thomson T., Wong B., Hoang V., Veljkovic E., Peck M. (2013). Bioinf. Biol. Insights.

[cit34] Park J., Schlage W., Frushour B., Talikka M., Toedter G., Gebel S., Deehan R., Veljkovic E., Westra J., Kogel U. (2013). J. Clin. Toxicol..

[cit35] Catlett N. L., Bargnesi A. J., Ungerer S., Seagaran T., Ladd W., Elliston K. O., Pratt D. (2013). BMC Bioinf..

[cit36] Boué S., Fields B., Hoeng J., Park J., Peitsch M. C., Schlage W. K., Talikka M., Binenbaum I., Bondarenko V., Bulgakov O. V., Cherkasova V., Diaz-Diaz N., Fedorova L., Guryanova S., Guzova J., Igorevna Koroleva G., Kozhemyakina E., Kumar R., Lavid N., Lu Q., Menon S., Ouliel Y., Peterson S. C., Prokhorov A., Sanders E., Schrier S., Schwaitzer Neta G., Shvydchenko I., Tallam A., Villa-Fombuena G., Wu J., Yudkevich I., Zelikman M. (2015). F1000Research.

[cit37] Subramanian A., Tamayo P., Mootha V. K., Mukherjee S., Ebert B. L., Gillette M. A., Paulovich A., Pomeroy S. L., Golub T. R., Lander E. S. (2005). Proc. Natl. Acad. Sci. U. S. A..

[cit38] Khatri P., Sirota M., Butte A. J. (2012). PLoS Comput. Biol..

[cit39] Thomson T. M., Sewer A., Martin F., Belcastro V., Frushour B. P., Gebel S., Park J., Schlage W. K., Talikka M., Vasilyev D. M. (2013). Toxicol. Appl. Pharmacol..

[cit40] Soste M., Hrabakova R., Wanka S., Melnik A., Boersema P., Maiolica A., Wernas T., Tognetti M., von Mering C., Picotti P. (2014). Nat. Methods.

[cit41] Smyth G. K. (2004). Stat. Appl. Genet. Mol. Biol..

[cit42] Kanehisa M., Goto S., Sato Y., Kawashima M., Furumichi M., Tanabe M. (2014). Nucleic Acids Res..

[cit43] Ackermann M., Strimmer K. (2009). BMC Bioinf..

[cit44] Wu D., Smyth G. K. (2012). Nucleic Acids Res..

[cit45] Wu D., Lim E., Vaillant F., Asselin-Labat M.-L., Visvader J. E., Smyth G. K. (2010). Bioinformatics.

[cit46] Frey B. J., Dueck D. (2007). Science.

[cit47] Majeed S., Frentzel S., Wagner S., Kuehn D., Leroy P., Guy P. A., Knorr A., Hoeng J., Peitsch M. C. (2014). Chem. Cent. J..

[cit48] Tilley A. E., Walters M. S., Shaykhiev R., Crystal R. G. (2015). Annu. Rev. Physiol..

[cit49] Stannard W., O'Callaghan C. (2006). J. Aerosol Med..

[cit50] Calderón-Garcidueñas L., Rodríguez-Alcaraz A., Villarreal-Calderón A., Lyght O., Janszen D., Morgan K. T. (1998). Toxicol. Sci..

[cit51] Harkema J. R., Carey S. A., Wagner J. G. (2006). Toxicol. Pathol..

[cit52] Davis M. A., Eldridge S. and Louden C., in Haschek and Rousseaux's Handbook of Toxicologic Pathology, ed. W. M. H. G. R. A. Wallig, Academic Press, Boston, 3rd edn, 2013, pp. 317–352, 10.1016/B978-0-12-415759-0.00010-8.

[cit53] Shimada T., Oda Y., Gillam E. M., Guengerich F. P., Inoue K. (2001). Drug Metab. Dispos..

[cit54] Zanetti F., Titz B., Sewer A., Sasso G. L., Scotti E., Schlage W. K., Mathis C., Leroy P., Majeed S., Torres L. O., Keppler B. R. (2017). Food Chem. Toxicol..

[cit55] Wooten J. B., Chouchane S. and McGrath T. E., in Cigarette Smoke and Oxidative Stress, Springer, 2006, pp. 5–46.

[cit56] Kogel U., Titz B., Schlage W. K., Nury C., Martin F., Oviedo A., Lebrun S., Elamin A., Guedj E., Trivedi K. (2016). Regul. Toxicol. Pharmacol..

[cit57] Tahmasbpour Marzony E., Ghanei M., Panahi Y. (2016). Exp. Lung Res..

[cit58] Pierrou S., Broberg P., O'donnell R. A., Pawłowski K., Virtala R., Lindqvist E., Richter A., Wilson S. J., Angco G., Möller S. (2007). Am. J. Respir. Crit. Care Med..

[cit59] Hackett N. R., Heguy A., Harvey B.-G., O'connor T. P., Luettich K., Flieder D. B., Kaplan R., Crystal R. G. (2003). Am. J. Respir. Cell Mol. Biol..

[cit60] Liberzon A., Birger C., Thorvaldsdóttir H., Ghandi M., Mesirov J. P., Tamayo P. (2015). Cell Syst..

[cit61] Lee J., Taneja V., Vassallo R. (2012). J. Dent. Res..

[cit62] Chung W. O. and Dommisch H., Antimicrobial Peptides of Skin and Oral Mucosa. In Innate Immune System of Skin and Oral Mucosa: Properties and Impact in Pharmaceutics, Cosmetics, and Personal Care Products, John Wiley and Sons, 2011, pp. 117–144.

[cit63] Mercer B. A., Wallace A. M., Brinckerhoff C. E., D'Armiento J. M. (2009). Am. J. Respir. Cell Mol. Biol..

[cit64] Pritchard C. C., Cheng H. H., Tewari M. (2012). Nat. Rev. Genet..

[cit65] Hu Y., Zhu Q., Tang L. (2014). PLoS One.

[cit66] Lerman G., Avivi C., Mardoukh C., Barzilai A., Tessone A., Gradus B., Pavlotsky F., Barshack I., Polak-Charcon S., Orenstein A. (2011). PLoS One.

[cit67] Warth S. C., Hoefig K. P., Hiekel A., Schallenberg S., Jovanovic K., Klein L., Kretschmer K., Ansel K. M., Heissmeyer V. (2015). EMBO J..

[cit68] Li X., Luo X., Han B., Duan F., Wei P., Chen Y. (2013). Br. J. Cancer.

[cit69] Cui L., Zhou H., Zhao H., Zhou Y., Xu R., Xu X., Zheng L., Xue Z., Xia W., Zhang B. (2012). BMC Cancer.

[cit70] Turcatel G., Rubin N., El-Hashash A., Warburton D. (2012). PLoS One.

[cit71] Park J.-K., Henry J. C., Jiang J., Esau C., Gusev Y., Lerner M. R., Postier R. G., Brackett D. J., Schmittgen T. D. (2011). Biochem. Biophys. Res. Commun..

[cit72] Rider C. F., Yamamoto M., Günther O. P., Hirota J. A., Singh A., Tebbutt S. J., Carlsten C. (2016). J. Allergy Clin. Immunol..

[cit73] Fry R. C., Rager J. E., Bauer R., Sebastian E., Peden D. B., Jaspers I., Alexis N. E. (2014). Am. J. Physiol.: Lung Cell. Mol. Physiol..

[cit74] Buas M. F., Onstad L., Levine D. M., Risch H. A., Chow W.-H., Liu G., Fitzgerald R. C., Bernstein L., Ye W., Bird N. C. (2015). PLoS One.

[cit75] Nebert D. W., Dalton T. P., Okey A. B., Gonzalez F. J. (2004). J. Biol. Chem..

[cit76] Franciosi L., Postma D. S., van den Berge M., Govorukhina N., Horvatovich P. L., Fusetti F., Poolman B., Lodewijk M. E., Timens W., Bischoff R., ten Hacken N. H. (2014). PLoS One.

[cit77] Titz B., Sewer A., Schneider T., Elamin A., Martin F., Dijon S., Luettich K., Guedj E., Vuillaume G., Ivanov N. V. (2015). J. Proteomics.

[cit78] Martin H.-J., Maser E. (2009). Chem.-Biol. Interact..

[cit79] Wang R., Wang G., Ricard M. J., Ferris B., Strulovici-Barel Y., Salit J., Hackett N. R., Gudas L. J., Crystal R. G. (2010). Chest J..

[cit80] Vanni H., Kazeros A., Wang R., Harvey B. G., Ferris B., De B. P., Carolan B. J., Hubner R. H., O'Connor T. P., Crystal R. G. (2009). Chest.

[cit81] Zhang X., Sebastiani P., Liu G., Schembri F., Zhang X., Dumas Y. M., Langer E. M., Alekseyev Y., O'Connor G. T., Brooks D. R. (2010). Physiol. Genomics.

[cit82] Titz B., Boue S., Phillips B., Talikka M., Vihervaara T., Schneider T., Nury C., Elamin A., Guedj E., Peck M. J., Schlage W. K., Cabanski M., Leroy P., Vuillaume G., Martin F., Ivanov N. V., Veljkovic E., Ekroos K., Laaksonen R., Vanscheeuwijck P., Peitsch M. C., Hoeng J. (2016). Toxicol. Sci..

[cit83] Kuilman T., Michaloglou C., Mooi W. J., Peeper D. S. (2010). Genes Dev..

[cit84] Tsuji T., Aoshiba K., Nagai A. (2004). Am. J. Respir. Cell Mol. Biol..

[cit85] Danielsson K., Ebrahimi M., Nylander E., Wahlin Y. B. and Nylander K., Alterations in Factors Involved in Differentiation and Barrier Function in the Epithelium in Oral and Genital Lichen Planus, 2017. 10.2340/00015555-2533 27599552

[cit86] Cabral A., Voskamp P., Cleton-Jansen A. M., South A., Nizetic D., Backendorf C. (2001). J. Biol. Chem..

[cit87] Andersen M. E., Krewski D. (2009). Toxicol. Sci..

[cit88] Tice R. R., Austin C. P., Kavlock R. J., Bucher J. R. (2013). Environ. Health Perspect..

[cit89] Richard A. M., Judson R. S., Houck K. A., Grulke C. M., Volarath P., Thillainadarajah I., Yang C., Rathman J., Martin M. T., Wambaugh J. F. (2016). Chem. Res. Toxicol..

[cit90] Kuczaj A. K., Nordlund M., Jayaraju S., Komen E., Krebs T., Peitsch M. C., Hoeng J. (2016). Appl. In Vitro Toxicol..

[cit91] Nordlund M., Kuczaj A. K. (2015). Comput. Syst. Toxicol..

[cit92] Wilmes A., Limonciel A., Aschauer L., Moenks K., Bielow C., Leonard M. O., Hamon J., Carpi D., Ruzek S., Handler A., Schmal O., Herrgen K., Bellwon P., Burek C., Truisi G. L., Hewitt P., Di Consiglio E., Testai E., Blaauboer B. J., Guillou C., Huber C. G., Lukas A., Pfaller W., Mueller S. O., Bois F. Y., Dekant W., Jennings P. (2013). J. Proteomics.

[cit93] Wilmes A., Bielow C., Ranninger C., Bellwon P., Aschauer L., Limonciel A., Chassaigne H., Kristl T., Aiche S., Huber C. G., Guillou C., Hewitt P., Leonard M. O., Dekant W., Bois F., Jennings P. (2015). Toxicol. In Vitro.

[cit94] Judson R. (2010). J. Toxicol. Environ. Health, Part B.

[cit95] Escher C., Reiter L., MacLean B., Ossola R., Herzog F., Chilton J., MacCoss M. J., Rinner O. (2012). Proteomics.

[cit96] Tarca A. L., Lauria M., Unger M., Bilal E., Boue S., Dey K. K., Hoeng J., Koeppl H., Martin F., Meyer P. (2013). Bioinformatics.

[cit97] Rhrissorrakrai K., Belcastro V., Bilal E., Norel R., Poussin C., Mathis C., Dulize R. H., Ivanov N. V., Alexopoulos L., Rice J. J. (2014). Bioinformatics.

[cit98] Poussin C., Belcastro V., Martin F., Boué S., Peitsch M. C., Hoeng J. (2017). Chem. Res. Toxicol..

[cit99] Boué S., Exner T., Ghosh S., Belcastro V., Dokler J., Page D., Boda A., Bonjour F., Hardy B., Vanscheeuwijck P., Hoeng J. and Peitsch M., Supporting evidence-based analysis for modified risk tobacco products through a toxicology data-sharing infrastructure, F1000Research, 2017, 6, 12. 29123642 10.12688/f1000research.10493.1 PMC5657032

[cit100] Edwards S. W., Tan Y.-M., Villeneuve D. L., Meek M., McQueen C. A. (2016). J. Pharmacol. Exp. Ther..

[cit101] Gonzalez-Suarez I., Martin F., Hoeng J., Peitsch M. C. (2016). Nutraceuticals: Efficacy, Safety and Toxicity.

[cit102] Szostak J., Martin F., Talikka M., Peitsch M. C., Hoeng J. (2016). Gene Regul. Syst. Biol..

[cit103] Flores M., Glusman G., Brogaard K., Price N. D., Hood L. (2013). Pers. Med..

