# Supplementary material for: Systems toxicology meta-analysis of in vitro assessment studies: biological impact of a candidate modified-risk tobacco product aerosol compared with cigarette smoke on human organotypic cultures of the aerodigestive tract
Source: Toxicol Res (Camb). 2017 May 29;6(5):631–53. doi: 10.1039/c7tx00047b (PMC6062142; doi:10.1039/c7tx00047b)
Supplement: Supplementary file 1 [file TX-006-C7TX00047B-s001.pdf]

Electronic Supplementary Material (ESI) for Toxicology Research.
This journal is © The Royal Society of Chemistry 2017

Supplementary Material

Systems Toxicology Meta-Analysis of In Vitro Assessment Studies: Biological Impact of a Candidate Modified-Risk Tobacco Product Aerosol Compared with Cigarette Smoke on Human Organotypic Cultures of the Aerodigestive Tract

A.R. Iskandar[a,#], B. Titz[a,#], A. Sewer[a,#], P. Leroy[a,#], T. Schneider[a], F. Zanetti[a], C. Mathis[a], A. Elamin[a], W. Schlage[b], F. Martin[a], M.C. Peitsch[a], and J. Hoeng[a,*]

[a] PMI R&D, Philip Morris Products S.A., Quai Jeanrenaud 5, CH-2000 Neuchâtel, Switzerland (Part of Philip Morris International group of companies).

[b] Biology consultant, Max-Baermann-Str. 21, 51429 Bergisch Gladbach, Germany

[#] A.R.I., B.T., A.S., and P.L. equally contributed to this manuscript

[*]Corresponding author:

Julia Hoeng, PhD

E-mail: julia.hoeng@pmi.com

Tel: +41 (58) 242 2214

Fax: +41 (58) 242 2811

Supplementary Figure 1. Gene set analysis (GSA) heatmap. Significance with respect to the treatment effect (Q2, compared with the air control) and dominant effects of individual gene sets (Q1) was assessed with Benjamini-Hochberg based FDR adjustment (FDR adj. p-value < 0.05). Gene sets are represented in the rows, exposure group comparisons versus sham in the columns, the gene set statistic is color-coded, and significance marked (see key).

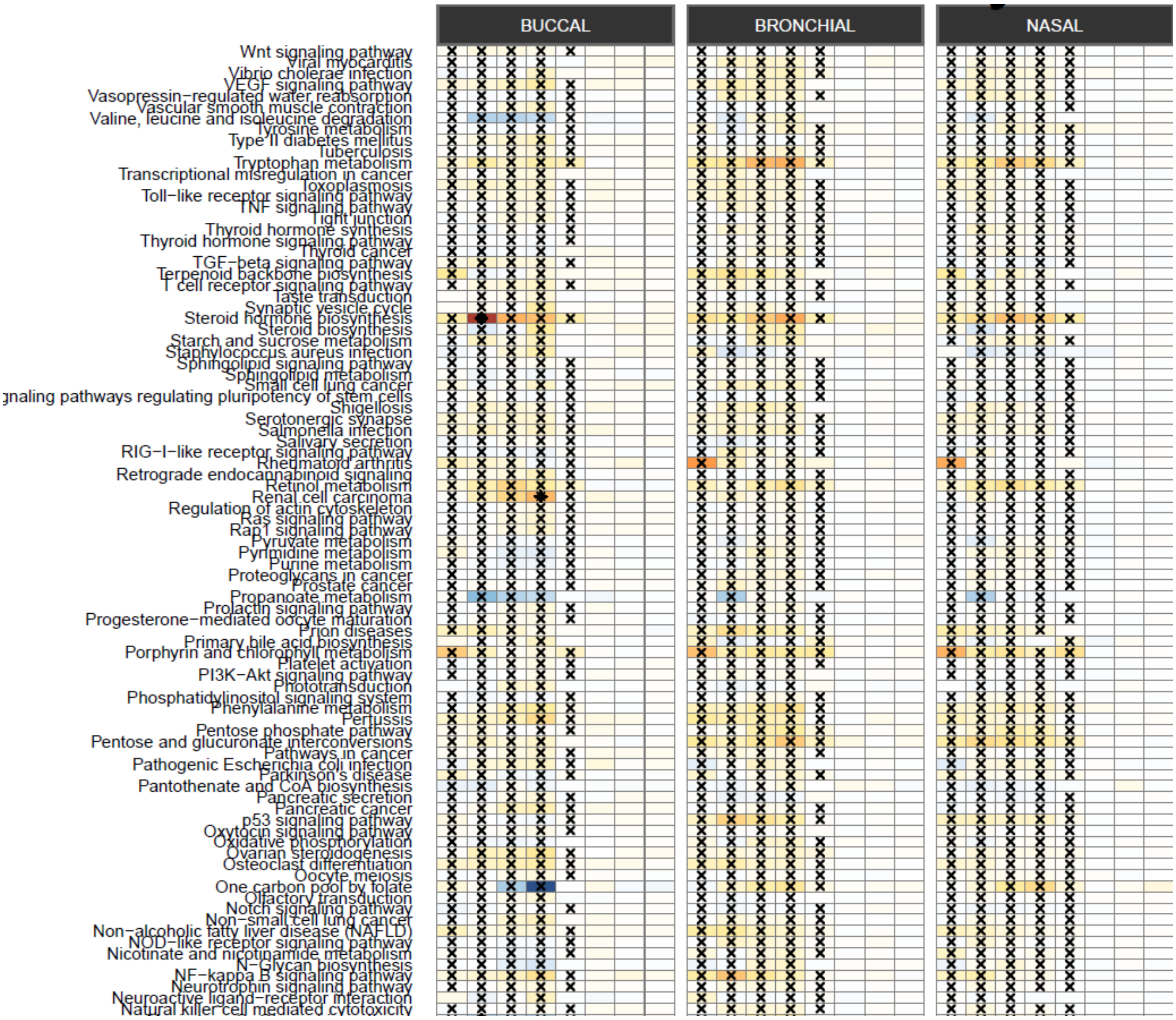

Figure continues

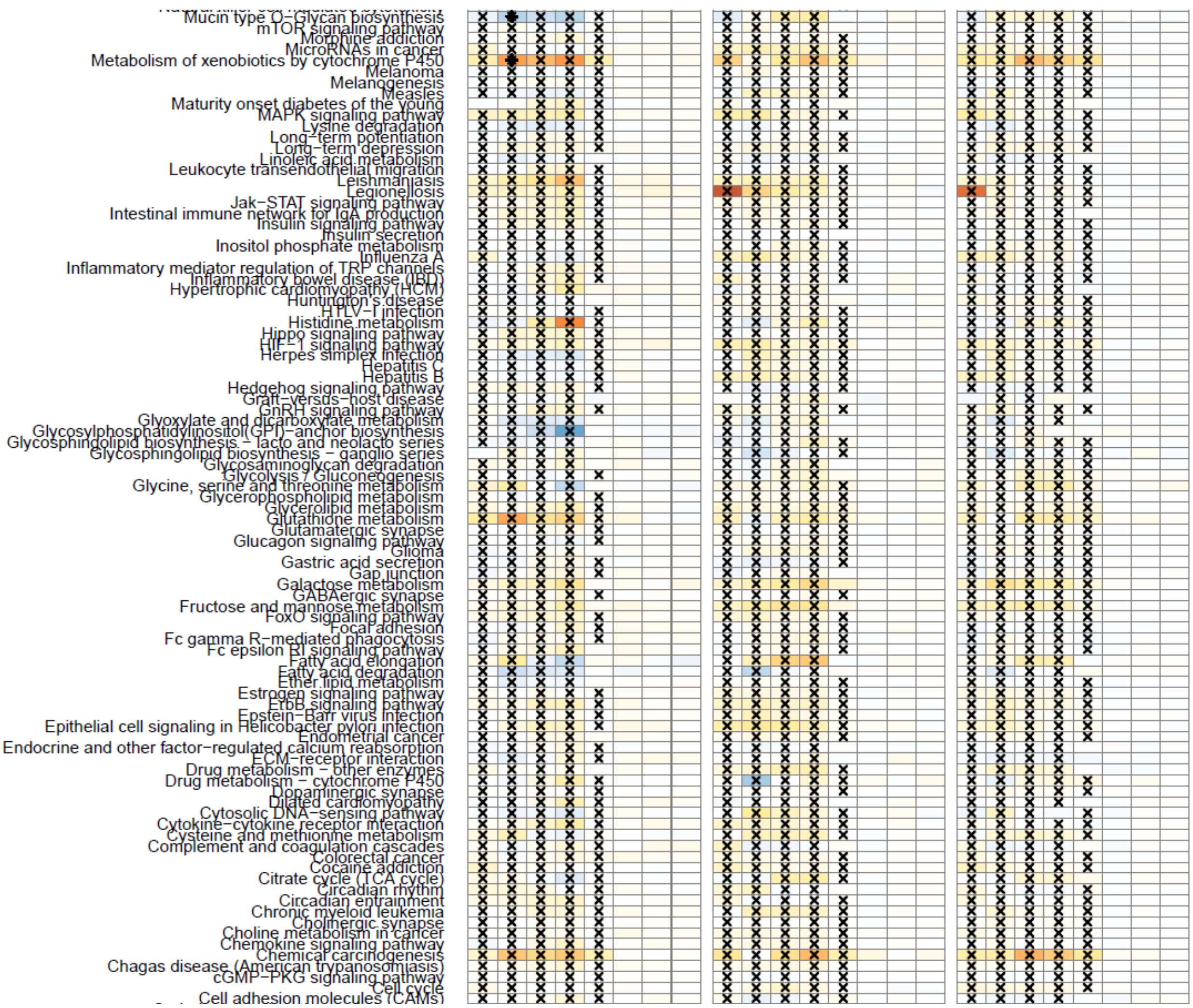

Figure continues

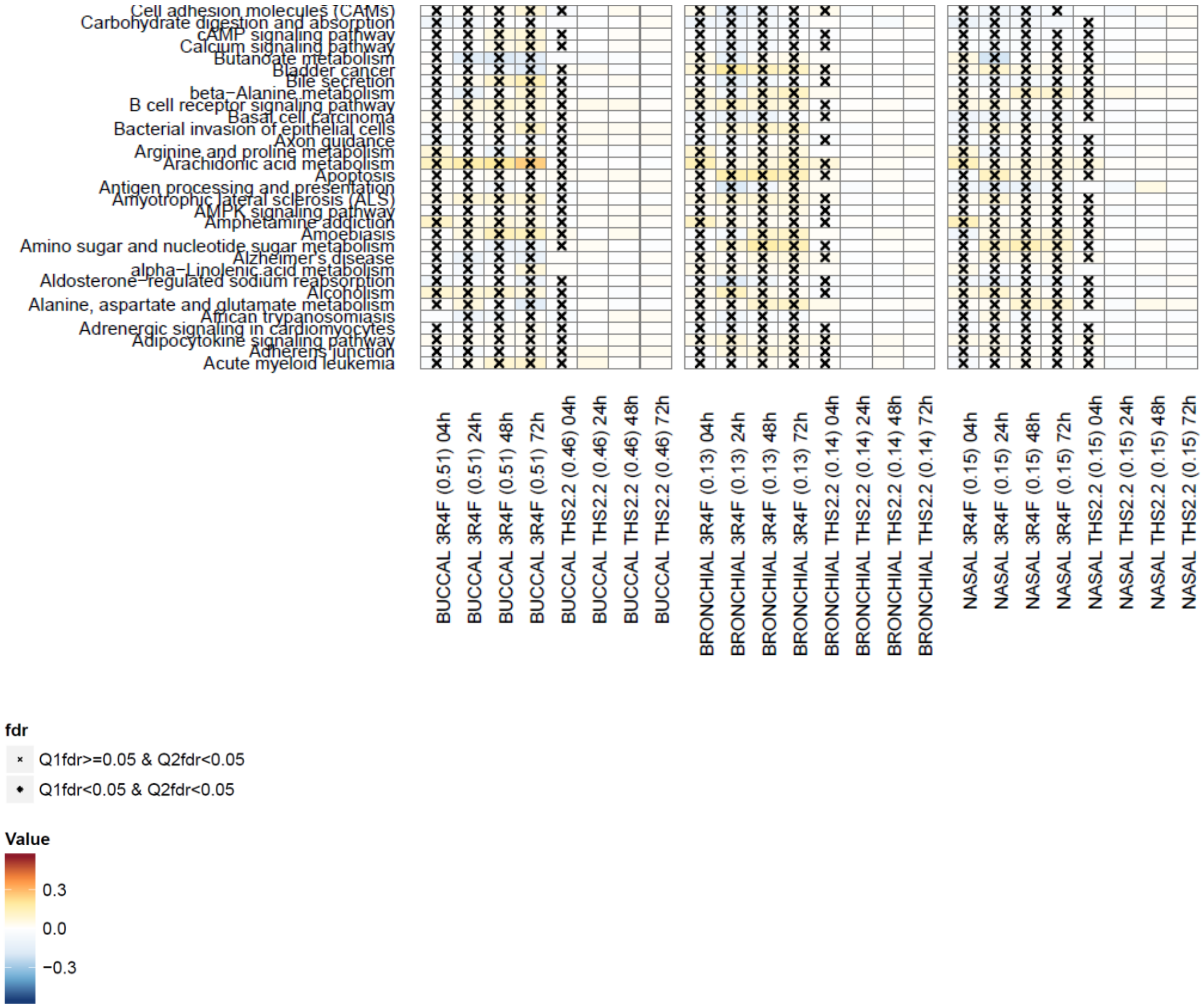
Cell adhesion molecules (CAMs)
Carbohydrate digestion and absorption
cAMP signaling pathway
Calcium signaling pathway
Butanoate metabolism
Bladder cancer
Bile secretion
beta−Alanine metabolism
B cell receptor signaling pathway
Basal cell carcinoma
Bacterial invasion of epithelial cells
Axon guidance
Arginine and proline metabolism
Arachidonic acid metabolism
Apoptosis
Antigen processing and presentation
Amyotrophic lateral sclerosis (ALS)
AMPK signaling pathway
Amphetamine addiction
Amoebiasis
Amino sugar and nucleotide sugar metabolism
Alzheimer's disease
alpha−Linolenic acid metabolism
Aldosterone−regulated sodium reabsorption
Alcoholism
Alanine, aspartate and glutamate metabolism
African trypanosomiasis
Adrenergic signaling in cardiomyocytes
Adipocytokine signaling pathway
Adherens junction
Acute myeloid leukemia
BUCCAL 3R4F (0.51) 04h
BUCCAL 3R4F (0.51) 24h
BUCCAL 3R4F (0.51) 48h
BUCCAL 3R4F (0.51) 72h
BUCCAL THS2.2 (0.46) 04h
BUCCAL THS2.2 (0.46) 24h
BUCCAL THS2.2 (0.46) 48h
BUCCAL THS2.2 (0.46) 72h
BRONCHIAL 3R4F (0.13) 04h
BRONCHIAL 3R4F (0.13) 24h
BRONCHIAL 3R4F (0.13) 48h
BRONCHIAL 3R4F (0.13) 72h
BRONCHIAL THS2.2 (0.14) 04h
BRONCHIAL THS2.2 (0.14) 24h
BRONCHIAL THS2.2 (0.14) 48h
BRONCHIAL THS2.2 (0.14) 72h
NASAL 3R4F (0.15) 04h
NASAL 3R4F (0.15) 24h
NASAL 3R4F (0.15) 48h
NASAL 3R4F (0.15) 72h
NASAL THS2.2 (0.15) 04h
NASAL THS2.2 (0.15) 24h
NASAL THS2.2 (0.15) 48h
NASAL THS2.2 (0.15) 72h
fdr
Q1fdr>=0.05 & Q2fdr<0.05
Q1fdr<0.05 & Q2fdr<0.05
Value
0.3
0.0
−0.3

Supplementary Figure 2. Heatmap of causal network enrichment results for all exposure group comparisons.

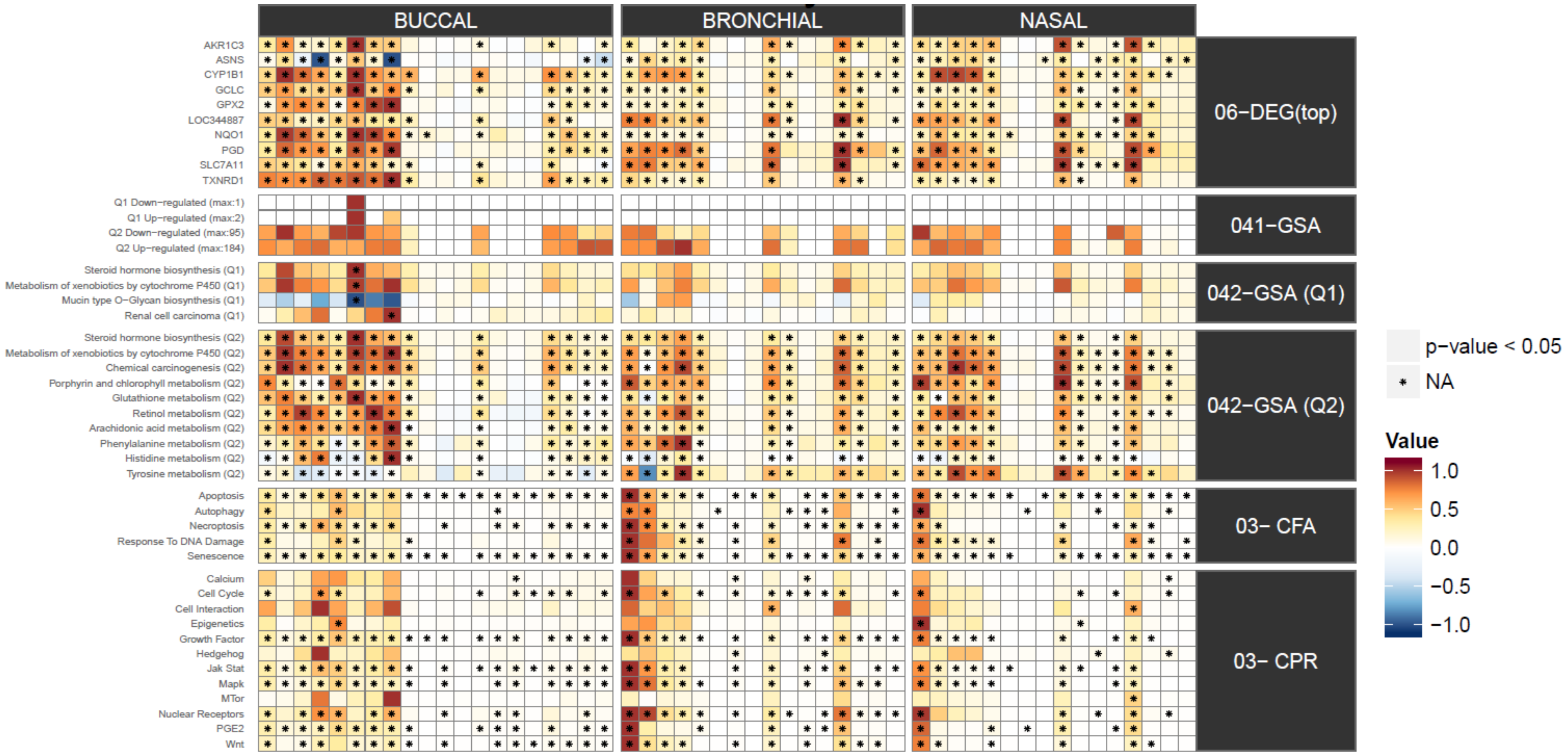

Figure continues

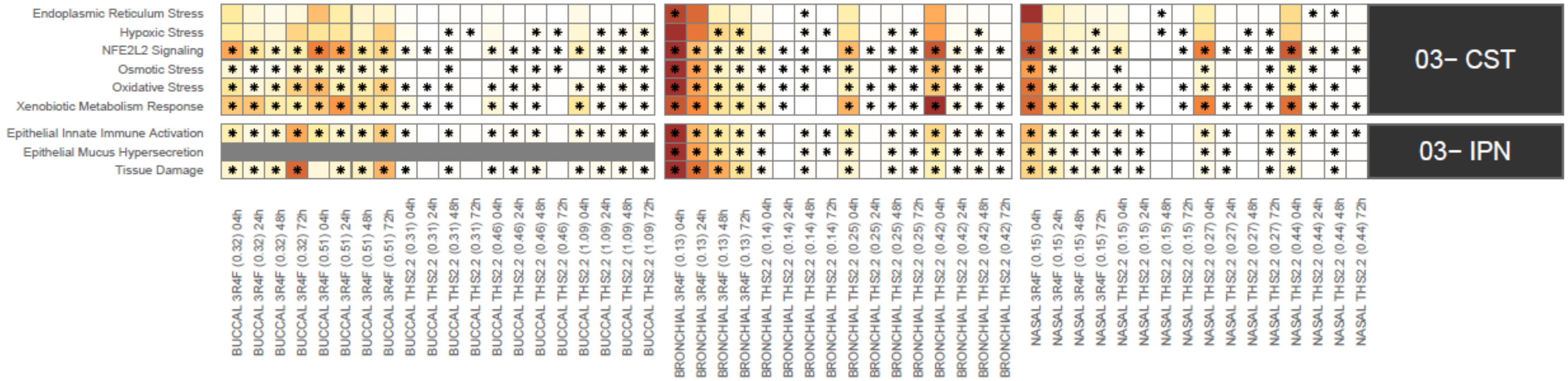

Endoplasmic Reticulum Stress
Hypoxic Stress
NFE2L2 Signaling
Osmotic Stress
Oxidative Stress
Xenobiotic Metabolism Response
Epithelial Innate Immune Activation
Epithelial Mucus Hypersecretion
Tissue Damage
03– CST
03– IPN
BUCCAL 3R4F (0.32) 04h
BUCCAL 3R4F (0.32) 24h
BUCCAL 3R4F (0.32) 48h
BUCCAL 3R4F (0.32) 72h
BUCCAL 3R4F (0.51) 04h
BUCCAL 3R4F (0.51) 24h
BUCCAL 3R4F (0.51) 48h
BUCCAL 3R4F (0.51) 72h
BUCCAL THS2.2 (0.31) 04h
BUCCAL THS2.2 (0.31) 24h
BUCCAL THS2.2 (0.31) 48h
BUCCAL THS2.2 (0.31) 72h
BUCCAL THS2.2 (0.46) 04h
BUCCAL THS2.2 (0.46) 24h
BUCCAL THS2.2 (0.46) 48h
BUCCAL THS2.2 (0.46) 72h
BUCCAL THS2.2 (1.09) 04h
BUCCAL THS2.2 (1.09) 24h
BUCCAL THS2.2 (1.09) 48h
BUCCAL THS2.2 (1.09) 72h
BRONCHIAL 3R4F (0.13) 04h
BRONCHIAL 3R4F (0.13) 24h
BRONCHIAL 3R4F (0.13) 48h
BRONCHIAL 3R4F (0.13) 72h
BRONCHIAL THS2.2 (0.14) 04h
BRONCHIAL THS2.2 (0.14) 24h
BRONCHIAL THS2.2 (0.14) 48h
BRONCHIAL THS2.2 (0.14) 72h
BRONCHIAL THS2.2 (0.25) 04h
BRONCHIAL THS2.2 (0.25) 24h
BRONCHIAL THS2.2 (0.25) 48h
BRONCHIAL THS2.2 (0.25) 72h
BRONCHIAL THS2.2 (0.42) 04h
BRONCHIAL THS2.2 (0.42) 24h
BRONCHIAL THS2.2 (0.42) 48h
BRONCHIAL THS2.2 (0.42) 72h
NASAL 3R4F (0.15) 04h
NASAL 3R4F (0.15) 24h
NASAL 3R4F (0.15) 48h
NASAL 3R4F (0.15) 72h
NASAL THS2.2 (0.15) 04h
NASAL THS2.2 (0.15) 24h
NASAL THS2.2 (0.15) 48h
NASAL THS2.2 (0.15) 72h
NASAL THS2.2 (0.27) 04h
NASAL THS2.2 (0.27) 24h
NASAL THS2.2 (0.27) 48h
NASAL THS2.2 (0.27) 72h
NASAL THS2.2 (0.44) 04h
NASAL THS2.2 (0.44) 24h
NASAL THS2.2 (0.44) 48h
NASAL THS2.2 (0.44) 72h

Supplementary Figure 3. Multianalyte profiling data for secreted pro-inflammatory mediators measured at various time point post-exposure and for all tested concentrations. The fold-changes relative to the respective sham groups are color coded. Grey cells indicate no measurement was conducted for the specific mediators.

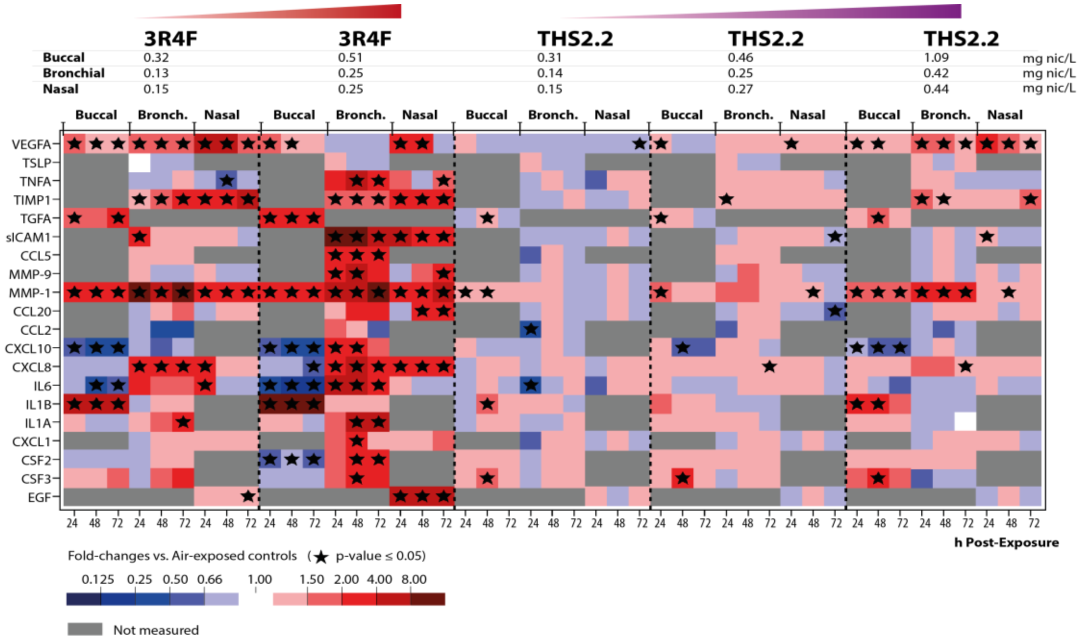

Supplementary Figure 4. Correlations between the secreted mediators and their corresponding mRNAs. The log2 fold-change of a secreted mediator at a given post-exposure time point (y-axis) is compared with the average log2 fold-change of the respective mRNA for the same and precedent times to account for accumulation of the secreted protein products (x-axis). All tested concentrations tested in *Supplementary Figure 3* are considered, see Figure 7C for the correlation for the comparable doses across tissues.

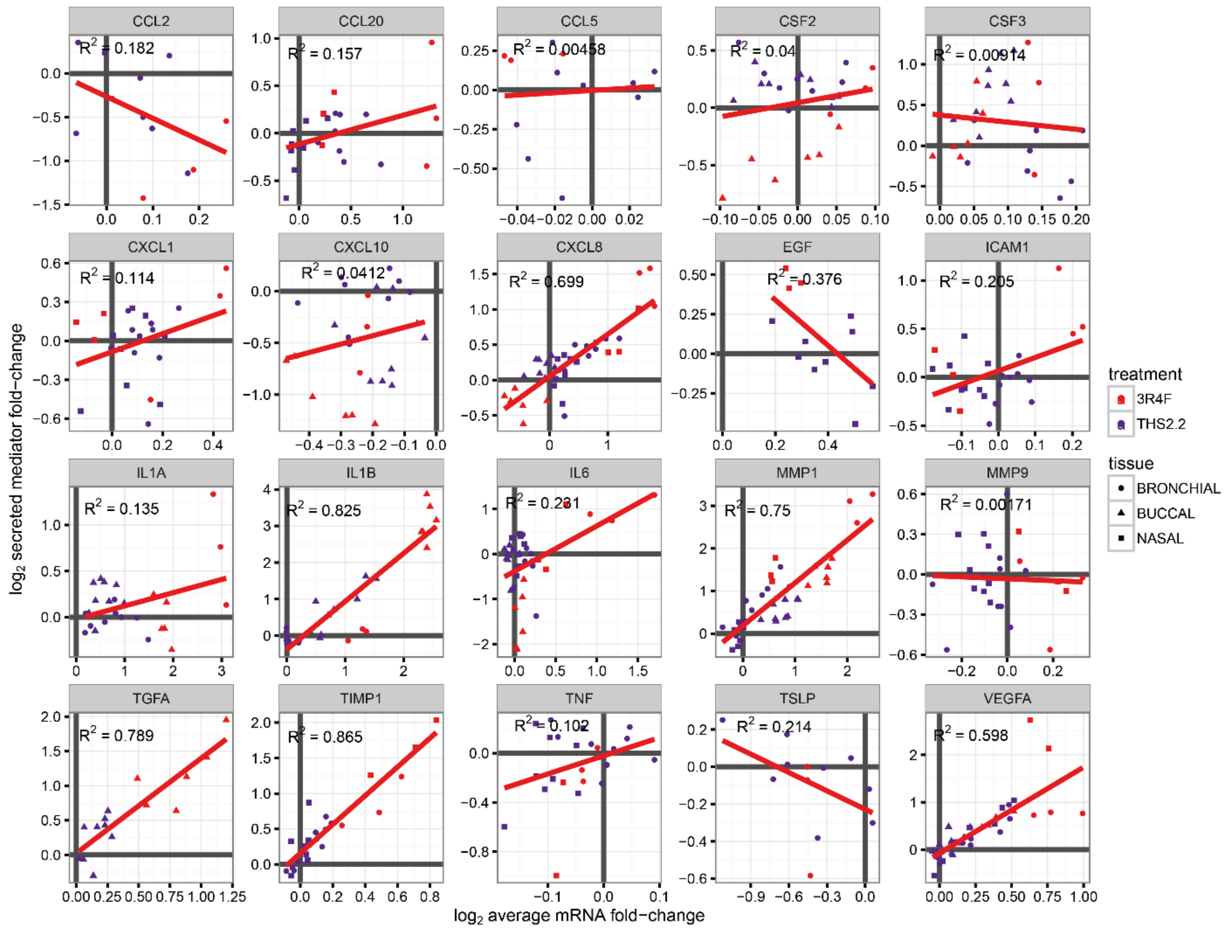

Supplementary Table 1: List of transitions used for quantification of the different protein targets by the PRM approach.

Target ID	Protein identifier	Peptide sequence	Modifications	Peptide type (endogenous/reference)	Precursor m/z	Precursor Charge state	Fragment ion	Fragment ion m/z	Fragment ion charge state
AKR1B10	sp|O60218|AK1BA_HUMAN	ALGVSNFSHFQIEK		Endogenous	526.27545	3	y10	618.80402	2
				Endogenous	526.27545	3	y5	664.36645	1
				Endogenous	526.27545	3	y7	888.45739	1
				Endogenous	526.27545	3	y8	1035.52581	1
				Endogenous	526.27545	3	y10	1236.60076	1
				Reference	528.94684	3	y10	622.81112	2
				Reference	528.94684	3	y5	672.38065	1
				Reference	528.94684	3	y7	896.47159	1
				Reference	528.94684	3	y8	1043.54001	1
				Reference	528.94684	3	y10	1244.61496	1
KRT14	sp|P02533|K1C14_HUMAN	APSTYGGGLSVSSSR		Endogenous	713.35205	2	y4	436.21504	1
				Endogenous	713.35205	2	y5	535.28345	1
				Endogenous	713.35205	2	y6	622.31548	1
				Endogenous	713.35205	2	y8	792.42101	1
				Endogenous	713.35205	2	y9	849.44247	1
				Endogenous	713.35205	2	y11	1069.52726	1
				Endogenous	713.35205	2	y12	1170.57494	1
				Endogenous	713.35205	2	y13	1257.60697	1
				Reference	718.35620	2	y4	446.22331	1
				Reference	718.35620	2	y5	545.29172	1
				Reference	718.35620	2	y6	632.32375	1
				Reference	718.35620	2	y8	802.42928	1
				Reference	718.35620	2	y9	859.45074	1
				Reference	718.35620	2	y11	1079.53553	1
				Reference	718.35620	2	y12	1180.58321	1
				Reference	718.35620	2	y13	1267.61524	1
FUCA1	sp|P04066|FUCO_HUMAN	DGLIVPIFQER		Endogenous	643.85876	2	y6	395.21632	2
				Endogenous	643.85876	2	y3	432.22012	1
				Endogenous	643.85876	2	y4	579.28854	1

				Endogenous	643.85876	2	y6	789.42536	1
				Endogenous	643.85876	2	y7	888.49378	1
				Endogenous	643.85876	2	y8	1001.57784	1
				Reference	648.86292	2	y6	400.22046	2
				Reference	648.86292	2	y3	442.22839	1
				Reference	648.86292	2	y4	589.29681	1
				Reference	648.86292	2	y6	799.43363	1
				Reference	648.86292	2	y7	898.50205	1
				Reference	648.86292	2	y8	1011.58611	1
CYP1A1	sp|P04798|CP1A1_HUMAN	IGSTPVVVLSGLDTIR		Endogenous	813.97491	2	y3	389.25069	1
				Endogenous	813.97491	2	y12	634.88227	2
				Endogenous	813.97491	2	y7	761.41519	1
				Endogenous	813.97491	2	y8	874.49926	1
				Endogenous	813.97491	2	y10	1072.63609	1
				Endogenous	813.97491	2	y12	1268.75726	1
				Reference	818.97900	2	y3	399.25896	1
				Reference	818.97900	2	y12	639.88640	2
				Reference	818.97900	2	y7	771.42346	1
				Reference	818.97900	2	y8	884.50753	1
				Reference	818.97900	2	y10	1082.64435	1
				Reference	818.97900	2	y12	1278.76553	1
P4HB	sp|P07237|PDIA1_HUMAN	YKPESEELTAER		Endogenous	726.35425	2	y10	580.77512	2
				Endogenous	726.35425	2	y5	589.33040	1
				Endogenous	726.35425	2	y6	718.37299	1
				Endogenous	726.35425	2	y8	934.44762	1
				Endogenous	726.35425	2	y9	1063.49021	1
				Endogenous	726.35425	2	y10	1160.54297	1
				Reference	731.35840	2	y10	585.77926	2
				Reference	731.35840	2	y5	599.33867	1
				Reference	731.35840	2	y6	728.38126	1
				Reference	731.35840	2	y8	944.45589	1
				Reference	731.35840	2	y9	1073.49848	1
				Reference	731.35840	2	y10	1170.55124	1
HSPA5	sp|P11021|GRP78_HUMAN	ITPSYVAFTPEGER		Endogenous	783.89355	2	y5	587.27837	1
				Endogenous	783.89355	2	y6	688.32604	1

				Endogenous	783.89355	2	y7	835.39446	1
				Endogenous	783.89355	2	y8	906.43157	1
				Endogenous	783.89355	2	y9	1005.49999	1
				Endogenous	783.89355	2	y10	1168.56331	1
				Endogenous	783.89355	2	y11	1255.59534	1
				Endogenous	783.89355	2	y12	1352.64811	1
				Reference	788.89771	2	y5	597.28664	1
				Reference	788.89771	2	y6	698.33431	1
				Reference	788.89771	2	y7	845.40273	1
				Reference	788.89771	2	y8	916.43984	1
				Reference	788.89771	2	y9	1015.50826	1
				Reference	788.89771	2	y10	1178.57158	1
				Reference	788.89771	2	y11	1265.60361	1
				Reference	788.89771	2	y12	1362.65638	1
G6PD	sp|P11413|G6PD_HUMAN	DGLLPENTFIVGYAR		Endogenous	832.93579	2	y4	466.24086	1
				Endogenous	832.93579	2	y5	565.30927	1
				Endogenous	832.93579	2	y11	633.82749	2
				Endogenous	832.93579	2	y6	678.39334	1
				Endogenous	832.93579	2	y7	825.46175	1
				Endogenous	832.93579	2	y8	926.50943	1
				Endogenous	832.93579	2	y9	1040.55236	1
				Endogenous	832.93579	2	y11	1266.64771	1
				Reference	837.93988	2	y4	476.24913	1
				Reference	837.93988	2	y5	575.31754	1
				Reference	837.93988	2	y11	638.83163	2
				Reference	837.93988	2	y6	688.40161	1
				Reference	837.93988	2	y7	835.47002	1
				Reference	837.93988	2	y8	936.51770	1
				Reference	837.93988	2	y9	1050.56063	1
				Reference	837.93988	2	y11	1276.65598	1
SCGB1A1	sp|P11684|UTER_HUMAN	LVDTLPQKPR		Endogenous	389.56790	3	y5	313.19265	2
				Endogenous	389.56790	3	y6	369.73468	2
				Endogenous	389.56790	3	y3	400.26668	1
				Endogenous	389.56790	3	y7	420.25852	2
				Endogenous	389.56790	3	y8	477.77199	2

				Endogenous	389.56790	3	y4	528.32526	1
				Endogenous	389.56790	3	y5	625.37802	1
				Endogenous	389.56790	3	y6	738.46208	1
				Endogenous	389.56790	3	y7	839.50976	1
				Endogenous	389.56790	3	y8	954.53671	1
				Reference	392.90399	3	y5	318.19678	2
				Reference	392.90399	3	y6	374.73882	2
				Reference	392.90399	3	y3	410.27495	1
				Reference	392.90399	3	y7	425.26265	2
				Reference	392.90399	3	y8	482.77613	2
				Reference	392.90399	3	y4	538.33353	1
				Reference	392.90399	3	y5	635.38629	1
				Reference	392.90399	3	y6	748.47035	1
				Reference	392.90399	3	y7	849.51803	1
				Reference	392.90399	3	y8	964.54498	1
PCNA	sp|P12004|PCNA_HUMAN	FSASGELGNGNIK		Endogenous	647.32532	2	y4	431.26126	1
				Endogenous	647.32532	2	y5	545.30419	1
				Endogenous	647.32532	2	y6	602.32565	1
				Endogenous	647.32532	2	y7	715.40971	1
				Endogenous	647.32532	2	y8	844.45231	1
				Endogenous	647.32532	2	y9	901.47377	1
				Endogenous	647.32532	2	y10	988.50580	1
				Endogenous	647.32532	2	y11	1059.54291	1
				Reference	651.33240	2	y4	439.27546	1
				Reference	651.33240	2	y5	553.31839	1
				Reference	651.33240	2	y6	610.33985	1
				Reference	651.33240	2	y7	723.42391	1
				Reference	651.33240	2	y8	852.46651	1
				Reference	651.33240	2	y9	909.48797	1
				Reference	651.33240	2	y10	996.52000	1
				Reference	651.33240	2	y11	1067.55711	1
NQO1	sp|P15559|NQO1_HUMAN	EGHLSPDIVAEQK		Endogenous	474.91229	3	y5	287.66339	2
				Endogenous	474.91229	3	y3	404.21397	1
				Endogenous	474.91229	3	y8	450.24528	2
				Endogenous	474.91229	3	y4	475.25109	1

				Endogenous	474.91229	3	y5	574.31950	1
				Reference	477.58371	3	y5	291.67049	2
				Reference	477.58371	3	y3	412.22817	1
				Reference	477.58371	3	y8	454.25237	2
				Reference	477.58371	3	y4	483.26529	1
				Reference	477.58371	3	y5	582.33370	1
GLB1	sp|P16278|BGAL_HUMAN	SLYPLTFIQVK		Endogenous	654.88171	2	y8	473.29203	2
				Endogenous	654.88171	2	y4	487.32386	1
				Endogenous	654.88171	2	y5	634.39227	1
				Endogenous	654.88171	2	y6	735.43995	1
				Endogenous	654.88171	2	y7	848.52402	1
				Endogenous	654.88171	2	y8	945.57678	1
				Reference	658.88885	2	y8	477.29913	2
				Reference	658.88885	2	y4	495.33806	1
				Reference	658.88885	2	y5	642.40647	1
				Reference	658.88885	2	y6	743.45415	1
				Reference	658.88885	2	y7	856.53822	1
				Reference	658.88885	2	y8	953.59098	1
ALDH3A1	sp|P30838|AL3A1_HUMAN	FDHILYTGSTGVGK		Endogenous	498.92444	3	y5	231.13955	2
				Endogenous	498.92444	3	y3	303.20268	1
				Endogenous	498.92444	3	y8	353.69014	2
				Endogenous	498.92444	3	y4	360.22415	1
				Endogenous	498.92444	3	y6	548.30385	1
				Endogenous	498.92444	3	y9	869.43632	1
				Reference	501.59583	3	y5	235.14665	2
				Reference	501.59583	3	y3	311.21688	1
				Reference	501.59583	3	y8	357.69724	2
				Reference	501.59583	3	y4	368.23834	1
				Reference	501.59583	3	y6	556.31805	1
				Reference	501.59583	3	y9	877.45052	1
SFN	sp|P31947|1433S_HUMAN	YLAEVATGDDK		Endogenous	591.28784	2	y3	189.08698	2
				Endogenous	591.28784	2	y7	353.17432	2
				Endogenous	591.28784	2	y3	377.16669	1
				Endogenous	591.28784	2	y4	434.18815	1
				Endogenous	591.28784	2	y9	453.21417	2

				Endogenous	591.28784	2	y6	606.27295	1
				Endogenous	591.28784	2	y7	705.34136	1
				Endogenous	591.28784	2	y8	834.38395	1
				Endogenous	591.28784	2	y9	905.42107	1
				Reference	595.29498	2	y3	193.09408	2
				Reference	595.29498	2	y7	357.18142	2
				Reference	595.29498	2	y3	385.18089	1
				Reference	595.29498	2	y4	442.20235	1
				Reference	595.29498	2	y9	457.22127	2
				Reference	595.29498	2	y6	614.28715	1
				Reference	595.29498	2	y7	713.35556	1
				Reference	595.29498	2	y8	842.39815	1
				Reference	595.29498	2	y9	913.43527	1
SPRR1A	sp|P35321|SPR1A_HUMAN	VPEPCPSTVTPAPAQQK	Carbamidomethyl [+57]	Endogenous	903.95636	2	y5	571.31984	1
				Endogenous	903.95636	2	y12	612.83278	2
				Endogenous	903.95636	2	y14	741.37448	2
				Endogenous	903.95636	2	y8	840.45739	1
				Endogenous	903.95636	2	y9	939.52581	1
				Endogenous	903.95636	2	y12	1224.65828	1
				Endogenous	903.95636	2	y13	1384.68893	1
				Endogenous	903.95636	2	y14	1481.74169	1
				Reference	907.96344	2	y5	579.33404	1
				Reference	907.96344	2	y12	616.83988	2
				Reference	907.96344	2	y14	745.38158	2
				Reference	907.96344	2	y8	848.47159	1
				Reference	907.96344	2	y9	947.54001	1
				Reference	907.96344	2	y12	1232.67248	1
				Reference	907.96344	2	y13	1392.70313	1
				Reference	907.96344	2	y14	1489.75589	1
p-CDKN1A/p21	sp|P38936|CDN1A_HUMAN	SGEQAEGSPGGPGDSQGR	Phospho [+80]	Endogenous	876.84467	2	y3	360.19899	1
				Endogenous	876.84467	2	y4	447.23102	1
				Endogenous	876.84467	2	y5	562.25797	1
				Endogenous	876.84467	2	y7	716.33219	1
				Endogenous	876.84467	2	y9	830.37512	1
				Endogenous	876.84467	2	y10	927.42788	1

				Endogenous	876.84467	2	y13	1280.49030	1
				Endogenous	876.84467	2	y14	1351.52741	1
				Reference	881.84882	2	y3	370.20726	1
				Reference	881.84882	2	y4	457.23929	1
				Reference	881.84882	2	y5	572.26623	1
				Reference	881.84882	2	y7	726.34046	1
				Reference	881.84882	2	y9	840.38339	1
				Reference	881.84882	2	y10	937.43615	1
				Reference	881.84882	2	y13	1290.49857	1
				Reference	881.84882	2	y14	1361.53568	1
CDKN1A/p21	sp|P38936|CDN1A_HUMAN	SGEQAEGSPGGPGDSQGR		Endogenous	836.86151	2	y4	447.23102	1
				Endogenous	836.86151	2	y9	830.37512	1
				Endogenous	836.86151	2	y10	927.42788	1
				Endogenous	836.86151	2	y11	1014.45991	1
				Endogenous	836.86151	2	y12	1071.48138	1
				Endogenous	836.86151	2	y13	1200.52397	1
				Endogenous	836.86151	2	y14	1271.56108	1
				Reference	841.86566	2	y4	457.23929	1
				Reference	841.86566	2	y9	840.38339	1
				Reference	841.86566	2	y10	937.43615	1
				Reference	841.86566	2	y11	1024.46818	1
				Reference	841.86566	2	y12	1081.48965	1
				Reference	841.86566	2	y13	1210.53224	1
				Reference	841.86566	2	y14	1281.56935	1
CDKN2A/p16-INK4a	sp|P42771|CDN2A_HUMAN	ALLEAGALPNAPNSYGR		Endogenous	857.44958	2	y6	693.33146	1
				Endogenous	857.44958	2	y7	764.36858	1
				Endogenous	857.44958	2	y8	878.41151	1
				Endogenous	857.44958	2	y9	975.46427	1
				Endogenous	857.44958	2	y10	1088.54833	1
				Endogenous	857.44958	2	y13	1287.64402	1
				Endogenous	857.44958	2	y14	1416.68662	1
				Reference	862.45367	2	y6	703.33973	1
				Reference	862.45367	2	y7	774.37685	1
				Reference	862.45367	2	y8	888.41977	1
				Reference	862.45367	2	y9	985.47254	1

				Reference	862.45367	2	y10	1098.55660	1
				Reference	862.45367	2	y13	1297.65229	1
				Reference	862.45367	2	y14	1426.69489	1
MKI67	sp|P46013|KI67_HUMAN	VEDAADSATKPENLSSK		Endogenous	587.95490	3	y3	321.17686	1
				Endogenous	587.95490	3	y11	581.30913	2
				Endogenous	587.95490	3	y12	638.82261	2
				Endogenous	587.95490	3	y14	709.85972	2
				Endogenous	587.95490	3	y15	767.37319	2
				Endogenous	587.95490	3	y7	774.39921	1
				Endogenous	587.95490	3	y8	902.49417	1
				Endogenous	587.95490	3	y9	1003.54185	1
				Endogenous	587.95490	3	y11	1161.61099	1
				Reference	590.62628	3	y3	329.19106	1
				Reference	590.62628	3	y11	585.31623	2
				Reference	590.62628	3	y12	642.82971	2
				Reference	590.62628	3	y14	713.86682	2
				Reference	590.62628	3	y15	771.38029	2
				Reference	590.62628	3	y7	782.41341	1
				Reference	590.62628	3	y8	910.50837	1
				Reference	590.62628	3	y9	1011.55605	1
				Reference	590.62628	3	y11	1169.62519	1
GCLC	sp|P48506|GSH1_HUMAN	SLFFPDEAINK		Endogenous	640.82971	2	y7	393.70324	2
				Endogenous	640.82971	2	y4	445.27691	1
				Endogenous	640.82971	2	y6	689.34645	1
				Endogenous	640.82971	2	y7	786.39921	1
				Endogenous	640.82971	2	y8	933.46762	1
				Endogenous	640.82971	2	y9	1080.53604	1
				Reference	644.83679	2	y7	397.71034	2
				Reference	644.83679	2	y4	453.29111	1
				Reference	644.83679	2	y6	697.36064	1
				Reference	644.83679	2	y7	794.41341	1
				Reference	644.83679	2	y8	941.48182	1
				Reference	644.83679	2	y9	1088.55024	1
AQP5	sp|P55064|AQP5_HUMAN	SFGPAVVMNR		Endogenous	539.27911	2	y7	393.71817	2
				Endogenous	539.27911	2	y3	420.20236	1

				Endogenous	539.27911	2	y4	519.27078	1
				Endogenous	539.27911	2	y8	843.45053	1
				Reference	544.28326	2	y7	398.72231	2
				Reference	544.28326	2	y3	430.21063	1
				Reference	544.28326	2	y4	529.27905	1
				Reference	544.28326	2	y8	853.45880	1
AQP4	sp|P55087|AQP4_HUMAN	GVWTQAFWK		Endogenous	561.79016	2	y3	480.26053	1
				Endogenous	561.79016	2	y4	551.29764	1
				Endogenous	561.79016	2	y6	780.40390	1
				Endogenous	561.79016	2	y7	966.48321	1
				Reference	565.79730	2	y3	488.27473	1
				Reference	565.79730	2	y4	559.31184	1
				Reference	565.79730	2	y6	788.41810	1
				Reference	565.79730	2	y7	974.49741	1
MUC5AC	sp|P98088|MUC5A_HUMAN	LYPAGSTIYR		Endogenous	570.80603	2	y8	432.73233	2
				Endogenous	570.80603	2	y3	451.26634	1
				Endogenous	570.80603	2	y4	552.31402	1
				Endogenous	570.80603	2	y5	639.34605	1
				Endogenous	570.80603	2	y6	696.36752	1
				Endogenous	570.80603	2	y7	767.40463	1
				Endogenous	570.80603	2	y8	864.45739	1
				Reference	575.81018	2	y8	437.73647	2
				Reference	575.81018	2	y3	461.27461	1
				Reference	575.81018	2	y4	562.32229	1
				Reference	575.81018	2	y5	649.35432	1
				Reference	575.81018	2	y6	706.37578	1
				Reference	575.81018	2	y7	777.41290	1
				Reference	575.81018	2	y8	874.46566	1
CYP1B1	sp|Q16678|CP1B1_HUMAN	VQAELDQVVGR		Endogenous	607.33038	2	y3	331.20883	1
				Endogenous	607.33038	2	y6	337.18502	2
				Endogenous	607.33038	2	y7	393.72705	2
				Endogenous	607.33038	2	y4	430.27724	1
				Endogenous	607.33038	2	y5	558.33582	1
				Endogenous	607.33038	2	y8	915.48942	1
				Endogenous	607.33038	2	y9	986.52654	1

				Reference	612.33453	2	y3	341.21710	1
				Reference	612.33453	2	y6	342.18915	2
				Reference	612.33453	2	y7	398.73119	2
				Reference	612.33453	2	y4	440.28551	1
				Reference	612.33453	2	y5	568.34409	1
				Reference	612.33453	2	y8	925.49769	1
				Reference	612.33453	2	y9	996.53480	1
DNAL1	sp|Q4LDG9|DNAL1_HUMAN	HSAENNWIEEATKR		Endogenous	562.27277	3	y3	404.26159	1
				Endogenous	562.27277	3	y7	423.73762	2
				Endogenous	562.27277	3	y4	475.29871	1
				Endogenous	562.27277	3	y5	604.34130	1
				Endogenous	562.27277	3	y6	733.38389	1
				Endogenous	562.27277	3	y7	846.46796	1
				Endogenous	562.27277	3	y8	1032.54727	1
				Reference	565.60889	3	y3	414.26986	1
				Reference	565.60889	3	y7	428.74175	2
				Reference	565.60889	3	y4	485.30698	1
				Reference	565.60889	3	y5	614.34957	1
				Reference	565.60889	3	y6	743.39216	1
				Reference	565.60889	3	y7	856.47623	1
				Reference	565.60889	3	y8	1042.55554	1
AQP3	sp|Q92482|AQP3_HUMAN	CGEMLHIR	Carbamidomethyl [+57]	Endogenous	508.24423	2	y3	213.13460	2
				Endogenous	508.24423	2	y4	269.67663	2
				Endogenous	508.24423	2	y6	399.71817	2
				Endogenous	508.24423	2	y3	425.26193	1
				Endogenous	508.24423	2	y4	538.34599	1
				Endogenous	508.24423	2	y6	798.42907	1
				Reference	513.24835	2	y3	218.13874	2
				Reference	513.24835	2	y4	274.68077	2
				Reference	513.24835	2	y6	404.72231	2
				Reference	513.24835	2	y3	435.27020	1
				Reference	513.24835	2	y4	548.35426	1
				Reference	513.24835	2	y6	808.43734	1
TP63	sp|Q9H3D4|P63_HUMAN	EFNEGQIAPPSHLIR		Endogenous	569.96527	3	y6	361.71903	2
				Endogenous	569.96527	3	y7	410.24541	2

				Endogenous	569.96527	3	y4	538.34599	1
				Endogenous	569.96527	3	y5	625.37802	1
				Endogenous	569.96527	3	y6	722.43078	1
				Endogenous	569.96527	3	y7	819.48355	1
				Reference	573.30139	3	y6	366.72316	2
				Reference	573.30139	3	y7	415.24955	2
				Reference	573.30139	3	y4	548.35426	1
				Reference	573.30139	3	y5	635.38629	1
				Reference	573.30139	3	y6	732.43905	1
				Reference	573.30139	3	y7	829.49182	1
MUC5B	sp|Q9HC84|MUC5B_HUMAN	SVVGDALEFGNSWK		Endogenous	754.87262	2	y4	534.26707	1
				Endogenous	754.87262	2	y6	738.35695	1
				Endogenous	754.87262	2	y7	867.39954	1
				Endogenous	754.87262	2	y10	1166.54766	1
				Endogenous	754.87262	2	y11	1223.56913	1
				Endogenous	754.87262	2	y12	1322.63754	1
				Reference	758.87970	2	y4	542.28127	1
				Reference	758.87970	2	y6	746.37115	1
				Reference	758.87970	2	y7	875.41374	1
				Reference	758.87970	2	y10	1174.56186	1
				Reference	758.87970	2	y11	1231.58333	1
				Reference	758.87970	2	y12	1330.65174	1
CGN	sp|Q9P2M7|CING_HUMAN	SHSQASLAGPGPVDPSNR		Endogenous	592.95898	3	y7	392.70103	2
				Endogenous	592.95898	3	y9	469.73815	2
				Endogenous	592.95898	3	y4	473.24667	1
				Endogenous	592.95898	3	y10	498.24888	2
				Endogenous	592.95898	3	y7	784.39479	1
				Endogenous	592.95898	3	y8	841.41626	1
				Reference	596.29504	3	y7	397.70517	2
				Reference	596.29504	3	y9	474.74228	2
				Reference	596.29504	3	y4	483.25494	1
				Reference	596.29504	3	y10	503.25301	2
				Reference	596.29504	3	y7	794.40306	1
				Reference	596.29504	3	y8	851.42453	1

Supplementary Table 2: Evaluated causal network models.

		Study		
Network Family	**Tissue Type**	**Buccal**	**Nasal**	**Bronchial**
CFA	Apoptosis	Y	Y	Y
CFA	Autophagy	Y	Y	Y
CFA	Necroptosis	Y	Y	Y
CFA	Response To DNA Damage	Y	Y	Y
CFA	Senescence	Y	Y	Y
CPR	Calcium	Y	Y	Y
CPR	Cell Cycle	Y	Y	Y
CPR	Cell Interaction	Y	Y	Y
CPR	Clock	Y	Y	Y
CPR	Epigenetics	Y	Y	Y
CPR	Growth Factor	Y	Y	Y
CPR	Hedgehog	Y	Y	Y
CPR	Hox	Y	Y	Y
CPR	Jak Stat	Y	Y	Y
CPR	Mapk	Y	Y	Y
CPR	MTor	Y	Y	Y
CPR	Notch	Y	Y	Y
CPR	Nuclear Receptors	Y	Y	Y
CPR	PGE2	Y	Y	Y
CPR	Wnt	Y	Y	Y
CST	Endoplasmic Reticulum Stress	Y	Y	Y
CST	Hypoxic Stress	Y	Y	Y
CST	NFE2L2 Signaling	Y	Y	Y
CST	Osmotic Stress	Y	Y	Y
CST	Oxidative Stress	Y	Y	Y
CST	Xenobiotic Metabolism Response	Y	Y	Y
IPN	Epithelial Innate Immune Activation	Y	Y	Y
IPN	Epithelial Mucus Hypersecretion	Not included	Y	Y
IPN	Tissue Damage	Y	Y	Y

Y = yes, network model was used for the analysis

CFA = cell fate; CPR = cell proliferation; CST = cell stress; IPN = inflammatory process network

Supplementary Table 3: Descriptive statistic of the measurement of nicotine in EXtrelut and deposited carbonyls.

	Nicotine			Carbonyl																	
	Nicotine (Extrelut)			Acetaldehyde			Acetone			Acrolein			Propionaldehyde			Crotonaldehyde			Methyl ethyl ketone		
**Exposure**	**N**	**M**	**SEM**	**N**	**M**	**SEM**	**N**	**M**	**SEM**	**N**	**M**	**SEM**	**N**	**M**	**SEM**	**N**	**M**	**SEM**	**N**	**M**	**SEM**
3R4F_Dose 1	38	0.13	0.004	36	1.26	0.070	36	0.75	0.021	36	0.09	0.003	36	0.07	0.004	36	0.07	0.002	36	0.19	0.005
3R4F_Dose 2	56	0.26	0.008	42	2.42	0.108	42	1.30	0.032	42	0.15	0.005	42	0.13	0.007	42	0.12	0.003	42	0.33	0.007
THS2.2_Dose 1	39	0.14	0.003	26	0.16	0.035	26	0.09	0.006	26	0.01	0.001	26	0.02	0.001	26	0.01	0.000	26	0.04	0.004
THS2.2_Dose 2	53	0.27	0.006	26	0.49	0.061	26	0.15	0.008	26	0.02	0.001	26	0.04	0.003	26	0.01	0.000	26	0.05	0.004
THS2.2_Dose 3	40	0.44	0.009	20	0.65	0.080	20	0.16	0.007	20	0.02	0.002	20	0.05	0.005	20	0.01	0.001	20	0.05	0.003

NA: not applicable

N = number of sampling throughout the study

M = mean

SEM = standard error of the mean
